# The Associations of Dietary Polyamines with Incident Type 2 Diabetes Mellitus: A Large Prospective Cohort Study

**DOI:** 10.3390/nu17010186

**Published:** 2025-01-04

**Authors:** Xiaohong Zhang, Mingxia Qian, Min Liu, Mengyao He, Fu-Rong Li, Liqiang Zheng

**Affiliations:** 1 School of Public Health, Shanghai Jiao Tong University School of Medicine, Shanghai 200025, China; zxhydx@shsmu.edu.cn (X.Z.); qianmx2000@126.com (M.Q.); 2 Department of Epidemiology, School of Public Health, China Medical University, Shenyang 110122, China; 17725165216@163.com (M.L.); myhe2016@163.com (M.H.); 3 School of Public Health and Emergency Management, Southern University of Science and Technology, Shenzhen 518055, China

**Keywords:** spermidine, spermine, putrescine, polyamines, T2DM, cox proportional hazards model, nonlinear association, prospective cohort study

## Abstract

Objectives: This study aimed to analyze the associations between dietary polyamine intake and incident T2DM. Methods: This prospective analysis included 168,137 participants from the UK Biobank who did not have T2DM at baseline. Dietary polyamines were calculated based on portion sizes of food items and a nutrient database. Incident T2DM was defined by hospital admissions with ICD10 codes E11–E14. Cox proportional hazard regression models and restricted cubic splines were used to examine the associations between dietary polyamine intake and incident T2DM. Results: During a median follow-up of 11.2 years (IQR, 11.8–13.2), 4422 (2.6%) participants developed T2DM. The average (SD) daily dietary intake was 10.5 (11.8) mg/day for spermidine, 4.3 (2.1) mg/day for spermine, and 12.7 (6.9) mg/day for putrescine. Compared to quintile 1, the multivariable-adjusted hazard ratios (95% CI) for quintiles 2–5 of dietary spermidine were 0.87 (0.79 to 0.96), 0.87 (0.79 to 0.96), 0.91 (0.82 to 0.99), and 0.96 (0.88 to 1.06); for dietary spermine, they were 1.01 (0.91 to 1.11), 1.03 (0.93 to 1.13), 1.07 (0.97 to 1.18), and 1.11 (1.01 to 1.23); and for dietary putrescine, they were 0.84 (0.76 to 0.92), 0.83 (0.79 to 0.91), 0.82 (0.74 to 0.90), and 0.87 (0.80 to 0.96). Conclusions: Higher dietary spermidine and putrescine were associated with a lower risk of T2DM, while higher dietary spermine appeared to be associated with a higher risk of T2DM. These findings suggest optimal levels of dietary polyamine intake and indicate that polyamines may be promising targets for nutritional interventions in the prevention and management of T2DM.

## 1. Introduction

With the aging population worldwide, the global prevalence of type 2 diabetes mellitus (T2DM), the most common form of diabetes, has reached epidemic levels, imposing significant socioeconomic burdens [1]. The International Diabetes Federation (IDF) estimated that 537 million adults aged 20–79 years (10.5% of the population) were living with diabetes mellitus in 2021 [1]. This number is projected to rise to 783 million by 2045, with 16% of the increase attributed to population aging [1]. Direct healthcare expenditures related to diabetes are nearing USD one trillion and are expected to surpass this figure by 2030 [1]. Various factors such as population aging, cigarette smoking, heavy alcohol consumption, sedentary lifestyle, increased body weight and waist circumference, low socioeconomic status, and pathological conditions like metabolic syndrome are associated with higher T2DM prevalence [2]. While certain foods and diets exhibit inhibitory effects on T2DM, traditional dietary guidelines often face participant adherence challenges in clinical practice. Therefore, novel nutritional interventions that focus on the molecular actions of food, rather than solely its nutritional value, are needed as a promising therapeutic strategy for T2DM management [3,4].

Polyamines, including spermidine, spermine, and putrescine, are compounds that contain two or more amine groups, enabling interactions with nucleic acids and proteins via negatively charged regions [5]. These polyamines are derived from exogenous sources, such as dietary intake [6] and gut microbiota [7], as well as endogenous sources, including intracellular de novo synthesis and the interconversion of other biomolecules [8]. Additionally, as de novo polyamine synthesis decreases with age, dietary sources become increasingly crucial for maintaining adequate polyamine levels [8]. Dietary polyamines are present in both animal- and plant-derived foods, either in free form or as conjugates [6]. The distribution of polyamines varies among foods, with meat being rich in spermine, while plant-derived foods have higher levels of putrescine and spermidine [9]. Spermidine and spermine are primarily found in raw plant and animal tissues, whereas putrescine can also be produced by fermentative or contaminating microorganisms [10,11]. While polyamines are widely recognized for their roles in cellular senescence and autophagy, as well as cell proliferation and differentiation, it is their antioxidant and anti-inflammatory properties that may play a more crucial role in preventing chronic diseases such as T2DM [10]. Dietary recommendations for T2DM prevention, such as the Mediterranean diet, often emphasize increased intake of polyamine-rich foods [12,13]. Therefore, it is hypothesized that increased polyamine intake may reduce the risk of T2DM [8,9,10,14,15,16,17].

The influence of polyamines on T2DM is complex and multifaceted [16,18]. Studies have shown that polyamine biosynthesis is crucial for the growth and differentiation of the pancreas in zebrafish [19] and that polyamine levels in pancreatic islets decrease with age in ob/ob mice [20]. Additionally, high glucose concentrations alter polyamine levels, and dysregulation of polyamine metabolism may impact glucose homeostasis in mice [21,22]. In individuals with T2DM, serum putrescine levels are significantly higher compared to those without diabetes, and serum putrescine and spermine levels correlate with glycosylated hemoglobin (HbA1c) and fasting insulin levels, respectively [23]. Serum spermidine also exhibits a nonlinear association with T2DM and fasting plasma glucose levels [24]. Furthermore, dietary spermidine negatively correlates with serum glucose, insulin, HbA1C levels, and the homeostatic model assessment of insulin resistance (HOMA-IR) index in both healthy and obese individuals without diabetes [25]. However, epidemiologic evidence regarding the impact of dietary polyamines on incident T2DM among the general population remains insufficient, highlighting the need for further research.

Drawing upon this evidence, our research aimed to investigate the association between dietary intake of spermidine, spermine, and putrescine, and the incidence of T2DM within a cohort of 168,137 participants from the UK Biobank.

## 2. Materials and Methods

### 2.1. Study Design and Population

The UK Biobank is a multicenter prospective population-based cohort that includes 502,490 participants aged 37 to 73 years from 22 assessment centers in the United Kingdom (England, Wales, and Scotland). Baseline data were collected between 2006 and 2010 and were linked to hospital and mortality records. At the initial visit, participants provided biological samples, completed a touch-screen questionnaire, underwent a computer-assisted face-to-face interview, and underwent a physical examination. Extensive information on sociodemographics, health behavior, and medical history was obtained via a touch-screen questionnaire and computer-assisted face-to-face interviews. Physical measures such as height and weight, as well as blood and urine samples, were collected by trained personnel following a standardized protocol. All participants provided written informed consent [26,27]. For this study, we excluded participants diagnosed with T2DM at baseline, those who were pregnant, those who did not complete a validated 24 h online dietary assessment, those without validated dietary spermidine/spermine/putrescine data, those with unreasonable total energy intake, and those missing covariate data. In total, 27,692 participants had a diabetes event at baseline, 148 were pregnant, 273,076 did not complete any validated dietary assessments, 14 were missing dietary spermidine/spermine/putrescine data, 1679 had unreasonable energy intake, and others were excluded due to missing covariate data, including BMI (516), region (81), smoking status (425), alcohol consumption (94), race/ethnicity (599), Townsend index (240), and MET score (29,789) (Figure S1).

### 2.2. Dietary Assessment

A web-based 24 h dietary instrument, the Oxford WebQ, was utilized to gather comprehensive dietary data. This instrument was validated against an interviewer-administered 24 h recall questionnaire. It effectively captured similar food items and provided comparable estimates of nutrient intake for a single day’s diet. As a self-administered tool that automatically estimates nutrient intake, it serves as a cost-effective method for assessing dietary intake in large-scale studies [28]. The Oxford WebQ captured information on the quantities of up to 206 widely consumed food items and 32 types of drinks consumed over the previous day [29]. Participants with valid email addresses were invited to complete a dietary questionnaire at baseline and were followed up for up to four times between April 2009 and June 2012 (cycle 1: February 2011 to April 2011; cycle 2: June 2011 to September 2011; cycle 3: October 2011 to December 2011; cycle 4: April 2012 to June 2012). A nutrient database for dietary spermidine, spermine, and putrescine (Table S6) was derived from published data and used to calculate their dietary spermidine, spermine, and putrescine intake based on portion sizes. This study analyzed the average dietary intake of these compounds in individuals who completed one or more diet assessments before developing T2DM. Among the 168,137 participants with at least one assessment, 64,646 completed one assessment, 38,839 completed two, 34,806 completed three, 25,126 completed four, and 4720 completed five assessments. Total energy intake was converted from kilojoules to kilocalories (kcal) by dividing by 4.184, with reasonable intake defined as 800–5000 kcal/day in men and 500–4000 kcal/day in women (including boundary values) [29].

### 2.3. Outcome Assessment

Incident T2DM events were assessed through hospital episodes in this study. These events were defined as hospital admissions with the *International Classification of Diseases, 10th Revision*, codes in the hospital records: E11–E14. Hospital admission data in England, Scotland, and Wales were collected up to 1 January 2022, and death data were linked from baseline up to the same date. The analysis of T2DM incidence was censored at the date of the first incident T2DM event, death, or 1 January 2022, whichever occurred first.

### 2.4. Assessment of Other Covariates

The potential confounding factors were informed by the prior literature [30]. These included self-reported baseline data on sex, age, race/ethnicity (white or non-white), regions (England, Northern Ireland, Republic of Ireland, Scotland, Wales, elsewhere), educational attainment (higher degree [college or university degree, or professional qualifications], any school degree [advanced levels, advanced subsidiary levels, ordinary levels, General Certificate of Secondary Education, Certificate of Secondary Education, or equivalent], vocational qualifications [National Vocational Qualification, Higher National Diploma, Higher National Certificate, or equivalent], other [none of the listed qualifications]), Townsend index (divided into quintiles 1–5, with higher scores indicating greater deprivation), smoking status (never, current, previous), alcohol consumption (never, current, previous), physical activity level (low [<600 metabolic equivalent (MET)—minutes/week], moderate [≥600 and <3000 MET—minutes/week], high [≥3000 MET—minutes/week]), and sleep duration. Body mass index (BMI) was calculated as weight (kg) divided by squared height (m), measured at baseline, and categorized as underweight [<18.5 kg/m^2^], healthy weight [18.5 to 25 kg/m^2^], overweight [25 to 30 kg/m^2^], or obese [≥30 kg/m^2^]. Hypertension at baseline was defined as a self-reported physician’s diagnosis or current use of antihypertensive medications. Cardiovascular disease at baseline was identified by self-reported history of heart attack, angina, or stroke. Hyperlipidemia was characterized by a self-reported physician’s diagnosis or use of lipid-lowering medications. Participants with diabetes at baseline, as determined by self-reported use of diabetes medications or physician’s diagnosis, were excluded from the study. Family history of diabetes was assessed through self-report at baseline. Table S1 provides detailed descriptions of these variables.

### 2.5. Statistical Analyses

Baseline participant characteristics were stratified based on quintiles 1–5 of dietary spermidine, spermine, and putrescine. Categorical variables were presented as counts and percentages, with differences assessed using Chi-Square tests. Normally distributed quantitative variables were summarized as means (SD) and compared using the Student *t*-test. Skewed distributed quantitative variables were described as medians (IQR) and differences were evaluated using non-parametric analysis.

Cox proportional hazards models were employed to calculate hazard ratios (HRs) and corresponding 95% confidence intervals (CIs) for each quintile of dietary spermidine, spermine, and putrescine, with quintile 1 serving as the reference category. Model 1 was adjusted for sociodemographic factors, including age, sex (men, women), race/ethnicity (white or non-white), and regions (England, Northern Ireland, Republic of Ireland, Scotland, Wales, elsewhere). Model 2 further accounted for educational level (higher degree, school degree, vocational qualifications, other) and Townsend index (quintiles 1–5). Model 3 additionally adjusted for smoking status (never, current, previous), drinking status (never, current, previous), physical activity level (low, moderate, high), sleep duration, and total energy intake (continuous). Model 4 further incorporated the family history of diabetes (yes/no), baseline hypertension (yes/no), baseline cardiovascular disease (yes/no), and baseline hyperlipidemia (yes/no). Model 5 (fully adjusted model) further included the BMI group (underweight, healthy weight, overweight, obese).

Restricted cubic spline (RCS) models with the same covariate specification of the fully adjusted Cox model and 4 knots were employed to assess the potential nonlinear associations between dietary spermidine, spermine, and putrescine and incident T2DM.

Stratified analyses were conducted as part of the additional investigations, evaluating the potential interactions of dietary spermidine, spermine, and putrescine with various demographic and health-related factors, including age group (<60, ≥60 years), sex (female, male), smoking status (never, previous, current), alcohol consumption (never, previous, current), Townsend index (quintiles 1–5), physical activity (low, moderate, high), family history of diabetes (yes/no), hypertension at baseline (yes/no), cardiovascular disease at baseline (yes/no), hyperlipidemia at baseline (yes/no), and BMI group (underweight/healthy, overweight, obesity). Multiplicative terms were incorporated into the Cox proportional hazards models to assess the potential heterogeneity of the associations between dietary spermidine, spermine, and putrescine and the risk of incident T2DM. Likelihood ratio tests were then performed to examine this heterogeneity across the subgroups.

The first sensitive analysis aimed to assess the robustness of the findings by imputing missing values of covariables. The second sensitive analysis was conducted using values of dietary spermidine, spermine, and putrescine ranging from 5% to 95%. The third sensitive analysis was conducted on those who completed two or more dietary assessments prior to the onset of T2DM. The fourth sensitive analysis focused on those with a follow-up period of two or more years, while the fifth sensitive analysis was limited to those with a follow-up period of five or more years. All analyses were carried out using R software (version 4.2.2, http://www.R-project.org, accessed on 4 November 2022). A two-tailed p value below 0.05 was the level of significance for the observed results.

## 3. Results

### 3.1. Study Participants

Baseline characteristics encompassing demographics, socioeconomic status, behavioral risk factors, health history, and dietary intake across quintiles of dietary spermidine concentrations are presented in Table 1. Our final sample consisted of 168,137 participants with a mean age of 55.8 years (SD, 8.0), an average daily dietary spermidine intake of 10.5 mg/day (SD, 11.8), and a mean sleep duration of 7.2 h (SD, 1.0). There were 4422 incident cases of T2DM (2.6%). Most participants were female (54.1%), born in England (81.0%), of white ethnicity (96.2%), with 60.1% reporting higher education, 53.4% reporting moderate physical activity levels, 57.0% being never smokers, and 94.3% being current drinkers. Furthermore, 80.2% had no family history of diabetes, 76.4% had no baseline hypertension, 96.0% had no baseline cardiovascular disease, 86.3% had no baseline hyperlipidemia, 39.3% were classified as overweight, and 32.2% were classified as obese. Apart from the Townsend index, baseline characteristics were generally consistent across quintiles of dietary spermidine. Notably, participants in the lower spermidine quintiles 1–2 were more often in the highest Townsend index quintile 5, while those in higher spermidine quintiles 3–5 were less represented in Townsend quintile 5. Baseline characteristics across quintiles of dietary spermine and putrescine concentrations are provided in Table S2 and Table S3, respectively. The average daily dietary spermine and putrescine intake in the cohort was 4.3 (SD, 2.1) mg/day and 12.7 (SD, 6.9) mg/day, respectively.

### 3.2. The Association of Dietary Polyamines with Incident T2DM

**Figure 1:**
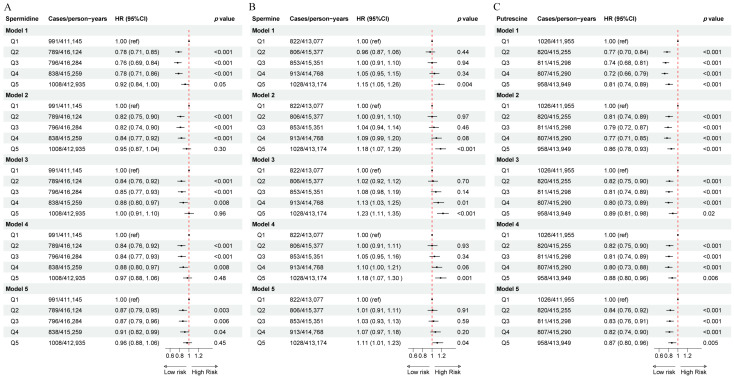
The associations of dietary spermidine, spermine, and putrescine with incident T2DM in the study population. (**A**) The association between dietary spermidine and incident T2DM. (**B**) The association between dietary spermine and incident T2DM. (**C**) The association between dietary putrescine and incident T2DM. Model 1 was adjusted for sociodemographic factors, including age, sex, race/ethnicity, and region. Model 2 was further adjusted for educational level and Townsend index. Model 3 was further adjusted for smoking status, drinking status, physical activity, sleep duration, and total energy intake. Model 4 was further adjusted for a family history of diabetes, hypertension at baseline, cardiovascular disease at baseline, and hyperlipidemia at baseline. Model 5 was further adjusted for the BMI group. Abbreviations: HR, hazard ratio. Q1–Q5, quintile 1–quintile 5. BMI, body mass index.

Over a median follow-up period of 11.2 years (IQR, 11.8–13.2), 4422 cases of T2DM were recorded. In quintiles 1–5 of dietary spermidine, the incidence of T2DM was 2.4, 1.9, 1.9, 2.0, and 2.4 cases per 1000 person-years, respectively. In the fully adjusted Cox proportional hazards model, compared with quintile 1, quintiles 2–4 of dietary spermidine were inversely associated with incident T2DM (quintile 2: HR = 0.87, 95% CI: 0.79 to 0.96, *p* = 0.003; quintile 3: HR = 0.87, 95% CI: 0.79 to 0.96, *p* = 0.006; quintile 4: HR = 0.91, 95% CI: 0.82 to 0.99, *p* = 0.04), while quintile 5 was not significantly associated (HR = 0.96, 95% CI: 0.88 to 1.06, *p* = 0.45) (Figure 1A). For dietary spermine, the incidence of T2DM across quintiles 1–5 was 2.0, 1.9, 2.1, 2.2, and 2.5 cases per 1000 person-years, respectively. In the fully adjusted Cox proportional hazards model, compared with quintile 1, quintile 5 was positively associated with incident T2DM (HR = 1.11, 95% CI: 1.01 to 1.23, *p* = 0.04), while quintiles 2–4 were not significantly associated (quintile 2: HR = 1.01, 95% CI: 0.91 to 1.11, *p* = 0.91; quintile 3: HR = 1.03, 95% CI: 0.93 to 1.13, *p* = 0.59; quintile 4: HR = 1.07, 95% CI: 0.97 to 1.18, *p* = 0.20) (Figure 1B). For dietary spermidine, the incidence of T2DM was 2.5, 2.0, 2.0, 1.9, and 2.3 cases per 1000 person-years, respectively. In the fully adjusted Cox proportional hazards model, compared with quintile 1, quintiles 2–5 were inversely associated with incident T2DM (quintile 2: HR = 0.84, 95% CI: 0.76 to 0.92, *p* < 0.001; quintile 3: HR = 0.83, 95% CI: 0.79 to 0.91, *p* < 0.001; quintile 4: HR = 0.82, 95% CI: 0.74 to 0.90, *p* < 0.001; quintile 5: HR = 0.87, 95% CI: 0.80 to 0.96, *p* = 0.005) (Figure 1C). The results were consistent across models 1, 2, 3, and 4, except that in model 3, quintile 4 of dietary spermine was positively associated with incident T2DM compared with quintile 1.

### 3.3. The Nonlinear Association of Dietary Polyamines with Incident T2DM

**Figure 2:**
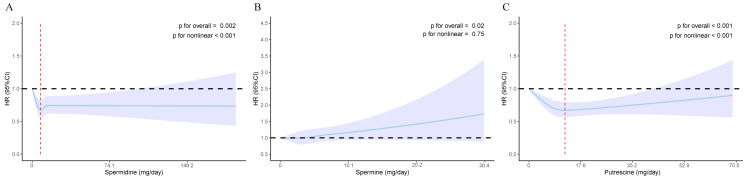
The nonlinear associations of dietary spermidine, spermine, and putrescine with incident T2DM in the study population. (**A**) The nonlinear association between dietary spermidine and incident T2DM. (**B**) The nonlinear association between dietary spermine and incident T2DM. (**C**) The nonlinear association between dietary putrescine and incident T2DM. The hazard ratio is indicated by solid lines and 95% CIs by shaded areas. The red dashed line indicates the value of dietary spermidine and putrescine at the inflection points (spermidine = 7.8 mg/day, putrescine = 12.2 mg/day). Models were adjusted for age, sex, race/ethnicity, regions, educational level, Townsend index, smoking status, drinking status, physical activity, sleep duration, total energy intake, family history of diabetes, hypertension at baseline, cardiovascular disease at baseline, hyperlipidemia at baseline, and BMI group. Abbreviations: T2DM, type 2 diabetes mellitus. HR, hazard ratio. CI, confidence interval. BMI, body mass index.

In fully adjusted RCS models, the associations of dietary spermidine and putrescine with incident T2DM were both L-shaped (Figure 2A,C). The *p*-values for overall association and nonlinearity of dietary spermidine were 0.002 and <0.001, respectively. For dietary putrescine, both *p*-values were <0.001. The inflection points in these L-shaped associations were at dietary spermidine and putrescine concentrations of 7.8 mg/day and 12.2 mg/day, respectively. However, the fully adjusted RCS model showed a linear association between dietary spermine and incident T2DM (Figure 2B), with *p*-values for overall association and nonlinearity being 0.02 and 0.75, respectively.

### 3.4. Stratified Analyses

Among women, never smokers, current alcohol consumers, participants in quintile 5 of the Townsend index, and those without baseline hypertension, cardiovascular disease, or hyperlipidemia, the association of serum spermidine and T2DM was similar to that observed in the overall cohort (Table 2). Notably, there was a statistically significant interaction between dietary spermidine and educational level (*p* for interaction = 0.03), while interactions with other covariates were not significant (*p* for interaction > 0.05).

For men aged 60 and above, previous smokers, current alcohol consumers, those in quintile 3 of the Townsend index, individuals with high physical activity, and those without baseline cardiovascular disease or hyperlipidemia, as well as overweight individuals, the associations of serum spermine with T2DM was consistent with that observed in the overall cohort (Table S4). Among participants under 60, those in quintile 2 of dietary spermine had a lower risk of T2DM (HR = 0.86, 95% CI: 0.74 to 0.99, *p* = 0.04). Conversely, participants aged 60 and above in quintile 2 of dietary spermine showed a higher risk of T2DM (HR = 1.16, 95% CI: 1.02 to 1.33, *p* = 0.03). Additionally, among men and participants aged 60 and above, as well as previous smokers, quintile 4 of dietary spermine was positively associated with T2DM. Overweight participants in quintile 3 of dietary spermine also showed a positive association with T2DM. Significant interactions were observed between dietary spermine and age group (*p* < 0.001), as well as race/ethnicity (*p* = 0.04), while interactions with other covariates were not significant (*p* for interaction > 0.05).

Among women, current alcohol consumers, participants in quintile 4 of the Townsend index, those of white ethnicity, individuals with baseline hypertension, and those without a family history of diabetes, cardiovascular disease, or hyperlipidemia, as well as overweight and obese individuals, the association between serum putrescine and T2DM was similar to that of the overall cohort (Table S5). There was a significant interaction between dietary putrescine and Townsend index (*p* for interaction = 0.03), while interactions with other covariates were not significant (*p* for interaction > 0.05).

### 3.5. Sensitivity Analyses

We conducted five sensitivity analyses to assess the robustness of the study findings. Imputing missing values of covariables maintained consistent associations and nonlinear patterns of dietary polyamines with incident T2DM, as observed in the overall study sample (Figures S2 and S3). Furthermore, we assessed the impact of optimal dietary polyamine levels on outcomes by using values ranging from the 5th to the 95th percentile of dietary spermidine, spermine, and putrescine. The results indicated generally similar associations and nonlinear patterns with incident T2DM as in the study sample, although quintile 4 of dietary spermidine in models 3 and 4, as well as quintiles 3 and 4 of dietary spermidine in the fully adjusted Cox model, were not statistically significant (Figures S4 and S5). Among participants with two or more dietary assessments, two or more years of follow-up, and five or more years of follow-up, the associations and nonlinear patterns of dietary polyamines with incident T2DM were largely consistent with the overall study sample, with some variations noted (Figures S6–S11). Specifically, in participants with two or more diet assessments, quintile 2 of dietary spermidine and quintile 5 of dietary spermine were not significantly associated with incident T2DM (Figure S6). Similarly, quintile 5 of dietary putrescine did not show a significant association with incident T2DM in models 3, 4, and the fully adjusted Cox model. The RCS model indicated that the overall *p*-value for dietary spermine was greater than 0.05 (Figure S7). Among participants with two or more years of follow-up, quintile 3 of dietary spermidine and quintile 5 of dietary spermine were not significantly associated with incident T2DM in the fully adjusted Cox model (Figure S8). Similarly, quintile 5 of dietary putrescine did not show a significant association with incident T2DM in models 3, 4, and the fully adjusted Cox model. Finally, among participants with five or more years of follow-up, dietary spermidine was not significantly associated with incident T2DM in the fully adjusted Cox model (Figure S10).

## 4. Discussion

This study, involving 168,137 participants from the UK Biobank with a median follow-up of 11.2 years (IQR, 11.8–13.2) from baseline, found that higher levels of spermidine and putrescine were associated with a lower risk of developing T2DM in a nonlinear fashion, while a higher level of dietary spermine was linked to a higher risk of T2DM in a linear fashion. These results suggest the presence of optimal levels of dietary polyamine intake.

The literature reveals discrepancies in the associations of serum polyamines with T2DM across different studies [23,24], with only one study reporting the beneficial impact of dietary spermidine on T2DM [25]. Most research on polyamines’ effect on T2DM has focused on animal models, revealing diverse findings. A study from 1974 noted insulin-like properties of spermidine and spermine but not putrescine [31]. In rat islets, prolonged exposure (24–48 h) to high glucose levels (20 mM) increased putrescine and spermidine production, but not spermine [32]. Exogenous spermine administration reduced body weight and fasting glucose levels and enhanced glucose tolerance in diet-induced obese mice [33]. Similarly, spermidine administration improved glycemic control and reduced HbA1c levels in diabetic rats [34]. Another study in the same rat model showed that arginine, putrescine, spermidine, or spermine administration protected β-cells [35].

Spermidine was mainly obtained from plant sources, with cereals, legumes, and soy derivatives as the richest sources. It was less common in animal products. Spermine is predominantly derived from animal products, with higher concentrations than spermidine in meat and derivatives, making it less prevalent in plant-derived foods. Putrescine was widely prevalent in both plant and animal foods but was especially abundant in fruits and vegetables. Its levels could increase due to spoilage bacteria [6]. Studies indicated that a plant-based diet, compared to an animal-based one, was associated with a reduced incidence of T2DM [36]. The observation was consistent with the conclusion of this study that higher levels of spermidine and putrescine in plant-based diets were associated with a lower risk of developing T2DM, whereas higher levels of spermine in animal-based diets were linked to a higher risk of T2DM. However, the content of polyamines in food was influenced by factors such as the plant’s origin, growing conditions, harvesting, and storage, as well as cooking techniques like boiling, grilling, or frying [6]. Additionally, the metabolism of polyamines in the human body is affected by aging, cellular function, and overall health outcomes [6]. Stratified and sensitivity analyses indicate that residual confounding such as educational level, age group, race/ethnicity, Townsend index, and BMI may contribute to some observed associations between dietary polyamines and incident T2DM. Additionally, causality between dietary polyamines and T2DM remains uncertain. Although the mechanisms underlying these associations warrant further elucidation, several studies offer valuable insights. For instance, spermidine, acting as a caloric restriction mimetic and natural autophagy inducer, can modulate the gut microbiota’s composition and function to alleviate metabolic syndrome induced by a high-fat diet through autophagy induction [25]. Exogenous spermine has been shown to mitigate diabetic cardiomyopathy by inhibiting ROS-p53-mediated downregulation of calcium-sensitive receptors and suppressing Wnt/β-catenin signaling [37]. Additionally, polyamines play a crucial role in modulating cellular inflammation by regulating macrophage polarization and T-cell differentiation [38,39,40]. Based on the preceding discussion, controlled dietary intervention studies manipulating polyamine intake are crucial for clarifying their effects on physiological and health markers. However, the influence of polyamines is complex and may be modulated by interactions with gut microbiota, other dietary components (such as aromatic herbs and spices and vitamin D), and other factors [41,42,43]. Therefore, a comprehensive understanding requires investigation beyond simple polyamine manipulation, encompassing studies examining the interplay between polyamines and these interacting elements. Additionally, exploring the biological mechanisms of polyamines’ impact on cellular function, aging, and disease would enhance our understanding of their significance as a dietary component.

The strengths of this study include a large sample size, repeated assessments through dietary questionnaires, and comprehensive data on covariates. This study is the first to investigate the association between dietary polyamine intake and the risk of T2DM. However, limitations necessitate acknowledgment. First, self-reported exposure data may introduce potential measurement errors; however, leveraging the average values from repeated dietary assessments enabled us to offer a rationalized range of dietary polyamine intake. Second, residual confounding may persist despite adjustments due to the observational nature of the study. Third, incident T2DM cases in this study were solely derived from hospital inpatient data, potentially leading to an underestimation of the true incidence. Last, the participants in this study were all individuals of European ancestry, potentially limiting the generalizability of the findings to other ethnic groups.

## 5. Conclusions

In this cohort from the United Kingdom, an intriguing observation emerged indicating that increased intake of dietary spermidine and putrescine was linked to a reduced risk of T2DM in a dose–response fashion, whereas elevated dietary spermine intake exhibited a linear trend correlating with higher T2DM risk. These findings suggest that polyamines could serve as a promising nutritional target for intervening in the incidence and management of T2DM.

## Figures and Tables

**Table 1:** Baseline characteristics of the study population according to quintiles of dietary spermidine *.

	Overall	Q1 (<6.2 mg/day)	Q2 (6.2~8.0 mg/day)	Q3 (8.0~9.8 mg/day)	Q4 (9.8~12.2 mg/day)	Q5 (>12.2 mg/day)	*p* Value
n	168,137	33,628	33,627	33,627	33,627	33,628	
Follow-up, years, median [IQR]	12.4 [11.8, 13.2]	12.4 [11.8, 13.2]	12.5 [11.9, 13.3]	12.5 [11.9, 13.3]	12.5 [11.9, 13.2]	12.4 [11.8, 13.2]	<0.001
Spermidine, mg/day	10.5 ± 11.8	4.6 ± 1.2	7.2 ± 0.5	8.9 ± 0.5	10.9 ± 0.7	21.1 ± 23.1	<0.001
Incident T2DM, n (%)							<0.001
No	163,715 (97.4)	32,637 (97.1)	32,838 (97.7)	32,831 (97.6)	32,789 (97.5)	32,620 (97.0)	
Yes	4422 (2.6)	991 (2.9)	789 (2.3)	796 (2.4)	838 (2.5)	1008 (3.0)	
Age	55.8 ± 8.0	54.5 ± 8.0	55.3 ± 7.9	56.0 ± 7.9	56.5 ± 7.9	56.6 ± 7.9	<0.001
Sex, n (%)							<0.001
Men	77,117 (45.9)	15,626 (46.5)	15,107 (44.9)	15,172 (45.1)	15,156 (45.1)	16,056 (47.7)	
Women	91,020 (54.1)	18,002 (53.5)	18,520 (55.1)	18,455 (54.9)	18,471 (54.9)	17,572 (52.3)	
Regions, n (%)							<0.001
England	136,222 (81.0)	26,950 (80.1)	27,143 (80.7)	27,394 (81.5)	27,522 (81.8)	27,213 (80.9)	
Northern Ireland	1146 (0.7)	238 (0.7)	240 (0.7)	219 (0.7)	233 (0.7)	216 (0.6)	
Republic of Ireland	1417 (0.8)	290 (0.9)	266 (0.8)	288 (0.9)	276 (0.8)	297 (0.9)	
Scotland	10,502 (6.2)	2062 (6.1)	2194 (6.5)	2131 (6.3)	2073 (6.2)	2042 (6.1)	
Wales	6198 (3.7)	1160 (3.4)	1234 (3.7)	1262 (3.8)	1300 (3.9)	1242 (3.7)	
Elsewhere	12,652 (7.5)	2928 (8.7)	2550 (7.6)	2333 (6.9)	2223 (6.6)	2618 (7.8)	
Race/ethnicity, n (%)							<0.001
White	161,683 (96.2)	31,761 (94.4)	32,420 (96.4)	32,628 (97.0)	32,568 (96.9)	32,306 (96.1)	
Non-white	6454 (3.8)	1867 (5.6)	1207 (3.6)	999 (3.0)	1059 (3.1)	1322 (3.9)	
Educational level, n (%)							<0.001
Higher degree	101,032 (60.1)	18,256 (54.3)	20,583 (61.2)	21,206 (63.1)	21,117 (62.8)	19,870 (59.1)	
Any school degree	47,138 (28.0)	10,634 (31.6)	9499 (28.2)	8884 (26.4)	8854 (26.3)	9267 (27.6)	
Vocational qualifications	7792 (4.6)	1791 (5.3)	1463 (4.4)	1405 (4.2)	1413 (4.2)	1720 (5.1)	
Other	12,175 (7.2)	2947 (8.8)	2082 (6.2)	2132 (6.3)	2243 (6.7)	2771 (8.2)	
Townsend index, n (%)							<0.001
Q1	33,641 (20.0)	6058 (18.0)	6735 (20.0)	7003 (20.8)	7082 (21.1)	6763 (20.1)	
Q2	33,642 (20.0)	6368 (18.9)	6532 (19.4)	6870 (20.4)	6982 (20.8)	6890 (20.5)	
Q3	33,607 (20.0)	6504 (19.3)	6632 (19.7)	6825 (20.3)	6793 (20.2)	6853 (20.4)	
Q4	33,619 (20.0)	6865 (20.4)	6799 (20.2)	6635 (19.7)	6591 (19.6)	6729 (20.0)	
Q5	33,628 (20.0)	7833 (23.3)	6929 (20.6)	6294 (18.7)	6179 (18.4)	6393 (19.0)	
Physical activity, n (%)							<0.001
Low	29,989 (17.8)	7129 (21.2)	6462 (19.2)	5883 (17.5)	5592 (16.6)	4923 (14.6)	
Moderate	89,800 (53.4)	17,479 (52.0)	18,295 (54.4)	18,537 (55.1)	18,263 (54.3)	17,226 (51.2)	
High	48,348 (28.8)	9020 (26.8)	8870 (26.4)	9207 (27.4)	9772 (29.1)	11,479 (34.1)	
Smoking status, n (%)							<0.001
Never	95,777 (57.0)	18,539 (55.1)	19,354 (57.6)	19,580 (58.2)	19,412 (57.7)	18,892 (56.2)	
Current	13,002 (7.7)	3734 (11.1)	2576 (7.7)	2219 (6.6)	2132 (6.3)	2341 (7.0)	
Previous	59,358 (35.3)	11,355 (33.8)	11,697 (34.8)	11,828 (35.2)	12,083 (35.9)	12,395 (36.9)	
Alcohol consumption, n (%)							<0.001
Never	4920 (2.9)	1112 (3.3)	911 (2.7)	878 (2.6)	915 (2.7)	1104 (3.3)	
Current	158,481 (94.3)	31,431 (93.5)	31,835 (94.7)	31,892 (94.8)	31,880 (94.8)	31,443 (93.5)	
Previous	4736 (2.8)	1085 (3.2)	881 (2.6)	857 (2.5)	832 (2.5)	1081 (3.2)	
Sleep duration, hours	7.2 ± 1.0	7.1 ± 1.1	7.2 ± 1.0	7.2 ± 1.0	7.2 ± 1.0	7.1 ± 1.1	<0.001
Family history of diabetes, n (%)							<0.001
No	134,902 (80.2)	26,653 (79.3)	27,151 (80.7)	27,077 (80.5)	27,088 (80.6)	26,933 (80.1)	
Yes	33,235 (19.8)	6975 (20.7)	6476 (19.3)	6550 (19.5)	6539 (19.4)	6695 (19.9)	
Hypertension, n (%)							<0.001
No	128,474 (76.4)	25,959 (77.2)	25,938 (77.1)	25,922 (77.1)	25,648 (76.3)	25,007 (74.4)	
Yes	39,663 (23.6)	7669 (22.8)	7689 (22.9)	7705 (22.9)	7979 (23.7)	8621 (25.6)	
Cardiovascular disease, n (%)							<0.001
No	161,475 (96.0)	32,353 (96.2)	32,365 (96.2)	32,333 (96.2)	32,340 (96.2)	32,084 (95.4)	
Yes	6662 (4.0)	1275 (3.8)	1262 (3.8)	1294 (3.8)	1287 (3.8)	1544 (4.6)	
Hyperlipidemia, n (%)							<0.001
No	145,054 (86.3)	29,250 (87.0)	29,198 (86.8)	29,037 (86.4)	29,037 (86.4)	28,532 (84.8)	
Yes	23,083 (13.7)	4378 (13.0)	4429 (13.2)	4590 (13.6)	4590 (13.6)	5096 (15.2)	
BMI group ^†^, n (%)							<0.001
Underweight	935 (0.6)	157 (0.5)	179 (0.5)	211 (0.6)	200 (0.6)	188 (0.6)	
Healthy weight	46,932 (27.9)	8766 (26.1)	9544 (28.4)	9789 (29.1)	9771 (29.1)	9062 (26.9)	
Overweight	66,137 (39.3)	13,094 (38.9)	13,516 (40.2)	13,372 (39.8)	13,268 (39.5)	12,887 (38.3)	
Obese	54,133 (32.2)	11,611 (34.5)	10,388 (30.9)	10,255 (30.5)	10,388 (30.9)	11,491 (34.2)	

Abbreviations: IQR, interquartile range. Q1–Q5, quintile 1–quintile 5. BMI, body mass index. * Unless otherwise indicated, data are expressed as mean ± SD. ^†^ BMI was calculated as weight in kilograms divided by height in meters squared. Underweight: BMI < 18.5 kg/m^2^; healthy weight: 18.5 ≤ BMI < 24 kg/m^2^; overweight: 24 ≤ BMI < 28 kg/m^2^; obese: BMI ≥ 28 kg/m^2^.

**Table 2:** The associations of dietary spermidine with incident T2DM among subgroups *.

	Cases/Person-Years	Q1 (<6.2 mg/day)	Q2 (6.2~8.0 mg/day)	Q3 (8.0~9.8 mg/day)	Q4 (9.8~12.2 mg/day)	Q5 (>12.2 mg/day)	*p* for Interaction
Age group							0.13
<60	2012/1,278,265	1.00	0.89 (0.78, 1.02)	0.83 (0.72, 0.96)	0.91 (0.79, 1.05)	0.88 (0.77, 1.02)	
≥60	2410/793,482	1.00	0.88 (0.77, 1.01)	0.95 (0.83, 1.09)	0.95 (0.83, 1.08)	1.09 (0.95, 1.24)	
Sex							0.72
Men	2670/942,905	1.00	0.91 (0.81, 1.03)	0.89 (0.79, 1.01)	0.96 (0.85, 1.09)	1.03 (0.91, 1.16)	
Women	1752/1,128,842	1.00	0.80 (0.69, 0.93)	0.85 (0.73, 0.98)	0.82 (0.71, 0.96)	0.87 (0.75, 1.01)	
Smoking status							0.67
Never	1976/1,188,596	1.00	0.86 (0.74, 0.99)	0.85 (0.74, 0.98)	0.84 (0.72, 0.97)	0.88 (0.76, 1.02)	
Current	511/157,194	1.00	0.78 (0.60, 1.03)	0.81 (0.61, 1.07)	1.08 (0.82, 1.42)	1.05 (0.80, 1.39)	
Previous	1935/725,956	1.00	0.90 (0.78, 1.04)	0.92 (0.79, 1.06)	0.95 (0.82, 1.09)	1.04 (0.90, 1.20)	
Alcohol consumption							0.55
Never	220/59,811	1.00	0.85 (0.56, 1.28)	0.73 (0.46, 1.13)	0.91 (0.60, 1.39)	0.91 (0.60, 1.39)	
Current	3962/1,955,145	1.00	0.87 (0.78, 0.96)	0.88 (0.79, 0.97)	0.89 (0.80, 0.98)	0.97 (0.88, 1.07)	
Previous	240/56,791	1.00	0.96 (0.64, 1.45)	1.04 (0.68, 1.59)	1.35 (0.90, 2.03)	1.06 (0.70, 1.60)	
Townsend index							0.07
Q1	670/420,573	1.00	1.05 (0.82, 1.35)	0.92 (0.71, 1.19)	1.00 (0.77, 1.29)	1.14 (0.88, 1.47)	
Q2	807/416,208	1.00	0.95 (0.80, 1.12)	0.91 (0.77, 1.08)	0.90 (0.76, 1.07)	1.05 (0.89, 1.25)	
Q3	849/414,799	1.00	0.83 (0.66, 1.04)	0.91 (0.73, 1.14)	1.05 (0.84, 1.31)	1.18 (0.95, 1.47)	
Q4	897/411,704	1.00	0.97 (0.79, 1.20)	1.04 (0.84, 1.28)	0.98 (0.79, 1.22)	0.89 (0.72, 1.11)	
Q5	1199/408,462	1.00	0.75 (0.63, 0.89)	0.73 (0.61, 0.87)	0.80 (0.67, 0.96)	0.80 (0.67, 0.96)	
Race/ethnicity							0.51
White	4101/1,994,658	1.00	0.86 (0.78, 0.95)	0.89 (0.80, 0.98)	0.91 (0.82, 1.00)	0.97 (0.88, 1.07)	
Non-white	321/77,089	1.00	0.92 (0.67, 1.28)	0.73 (0.50, 1.06)	0.92 (0.65, 1.31)	0.92 (0.64, 1.31)	
Physical activity							0.63
Low	1118/367,879	1.00	0.91(0.76, 1.09)	0.91 (0.75, 1.09)	0.92 (0.77, 1.11)	0.85 (0.70, 1.03)	
Moderate	2208/1,109,518	1.00	0.85 (0.75, 0.98)	0.90 (0.78, 1.03)	0.89 (0.78, 1.03)	0.99 (0.86, 1.14)	
High	1096/594,350	1.00	0.84 (0.69, 1.03)	0.80 (0.65, 0.98)	0.91 (0.75, 1.11)	1.00 (0.83, 1.21)	
Educational level							0.03
Higher degree	2166/1,252,991	1.00	0.84 (0.73, 0.96)	0.83 (0.72, 0.95)	0.82 (0.72, 0.95)	1.01 (0.88, 1.16)	
Any school degree	1280/579,043	1.00	0.97 (0.81, 1.15)	0.92 (0.77, 1.10)	1.05 (0.88, 1.25)	0.97 (0.81, 1.17)	
Vocational qualifications	314/145,227	1.00	1.14 (0.80, 1.62)	1.06 (0.73, 1.53)	1.09 (0.75, 1.58)	1.23 (0.86, 1.75)	
Other	662/94,487	1.00	0.67 (0.52, 0.87)	0.86 (0.68, 1.08)	0.84 (0.66, 1.07)	0.74 (0.58, 0.94)	
Family history of diabetes							0.54
No	2932/1,666,274	1.00	0.86 (0.77, 0.97)	0.89 (0.79, 1.00)	0.88 (0.78, 0.99)	0.99 (0.88, 1.11)	
Yes	1490/405,473	1.00	0.87 (0.74, 1.02)	0.84 (0.71, 0.99)	0.95 (0.81, 1.12)	0.92 (0.78, 1.09)	
Hypertension							0.31
No	2203/159,4562	1.00	0.84 (0.74, 0.96)	0.87 (0.76, 0.99)	0.83 (0.73, 0.96)	0.98 (0.86, 1.13)	
Yes	2219/477,185	1.00	0.89 (0.78, 1.02)	0.88 (0.77, 1.01)	0.97 (0.85, 1.12)	0.95 (0.83, 1.09)	
Cardiovascular disease							0.32
No	3868/1,993,952	1.00	0.84 (0.76, 0.93)	0.87 (0.79, 0.97)	0.90 (0.81, 0.99)	0.94 (0.84, 1.04)	
Yes	554/77,795	1.00	1.07 (0.82, 1.40)	0.87 (0.66, 1.16)	0.96 (0.72, 1.27)	1.18 (0.90, 1.53)	
Hyperlipidemia							0.15
No	2930/1,797,116	1.00	0.81 (0.72, 0.91)	0.84 (0.75, 0.94)	0.85 (0.76, 0.95)	0.87 (0.78, 0.98)	
Yes	1492/274,631	1.00	1.00 (0.84, 1.18)	0.97 (0.82, 1.15)	1.04 (0.88, 1.23)	1.17 (0.99, 1.38)	
BMI group ^†^							0.52
Underweight/healthy weight	338/597,623	1.00	0.66 (0.46, 0.94)	0.78 (0.55, 1.10)	0.78 (0.56, 1.11)	0.95 (0.67, 1.35)	
Overweight	1086/819,249	1.00	0.95 (0.78, 1.15)	0.91 (0.75, 1.11)	0.88 (0.72, 1.08)	1.12 (0.92, 1.36)	
Obese	2998/654,876	1.00	0.86 (0.77, 0.97)	0.87 (0.78, 0.98)	0.93 (0.83, 1.04)	0.92 (0.81, 1.03)	

* Models were adjusted for age, sex, race/ethnicity, regions, educational level, Townsend index, smoking status, drinking status, physical activity, sleep duration, total energy intake, family history of diabetes, hypertension at baseline, cardiovascular disease at baseline, hyperlipidemia at baseline, and BMI group. ^†^ BMI was calculated as weight in kilograms divided by height in meters squared. Underweight: BMI < 18.5 kg/m^2^; healthy weight: 18.5 ≤ BMI < 24 kg/m^2^; overweight: 24 ≤ BMI < 28 kg/m^2^; obese: BMI ≥ 28 kg/m^2^.

## Data Availability

Researchers can apply to use the UK Biobank resource and access the data used. No additional data are available.

## Supplementary Materials

The following supporting information can be downloaded at https://www.mdpi.com/article/10.3390/nu17010186/s1: Figure S1: Participant flow chart of the study; Table S1: Derivation of variables used in analysis from the UK Biobank questionnaire and interviews; Table S2: Baseline characteristics of the study population according to quintiles of dietary spermine; Table S3: Baseline characteristics of the study population according to quintiles of dietary putrescine; Table S4: The associations of dietary spermine with incident T2DM among subgroups; Table S5: The associations of dietary putrescine with incident T2DM among subgroups; Figure S2: The associations of dietary polyamine with incident T2DM among participants after imputing missing values of covariables; Figure S3: The nonlinear associations of dietary polyamine with incident T2DM among participants after imputing missing values of covariables; Figure S4: The associations of dietary polyamine with incident T2DM after deleting extreme values of dietary polyamines; Figure S5: The nonlinear associations of dietary polyamine with incident T2DM after deleting extreme values of dietary polyamines; Figure S6: The associations of dietary polyamine with incident T2DM among participants with two or more diet assessments; Figure S7: The nonlinear associations of dietary polyamine with incident T2DM among participants with two or more diet assessments; Figure S8: The associations of dietary polyamine with incident T2DM among participants with two or more years of follow-up; Figure S9: The nonlinear associations of dietary polyamine with incident T2DM among participants with two or more years of follow-up; Figure S10: The associations of dietary polyamine with incident T2DM among participants with five or more years of follow-up; Figure S11: The nonlinear associations of dietary polyamine with incident T2DM among participants with five or more years of follow-up. Table S6: Nutrient Database for Polyamine Intake [10,11,44,45,46,47,48,49,50,51,52,53,54,55].

## Abbreviations

T2DM	type 2 diabetes mellitus
SD	standard deviation
IQR	interquartile range
HR	hazard ratio
CI	confidence interval
HbA1C	glycosylated hemoglobin A1c
HOMA-IR	homeostatic model assessment of insulin resistance
ROS	reactive oxygen species
Wnt	Wingless/Integrated
