# Supplementary material for: The Associations of Dietary Polyamines with Incident Type 2 Diabetes Mellitus: A Large Prospective Cohort Study
Source: Nutrients. 2025 Jan 4;17(1):186. doi: 10.3390/nu17010186 (PMC11722915; doi:10.3390/nu17010186)
Supplement: Supplementary file 1 [file nutrients-17-00186-s001.zip › nutrients-3371155-supplementary.pdf]

The associations of dietary polyamines with incident type 2 diabetes mellitus: a large prospective cohort study

Supplemental materials

Figure S1. Participants flow chart of the study

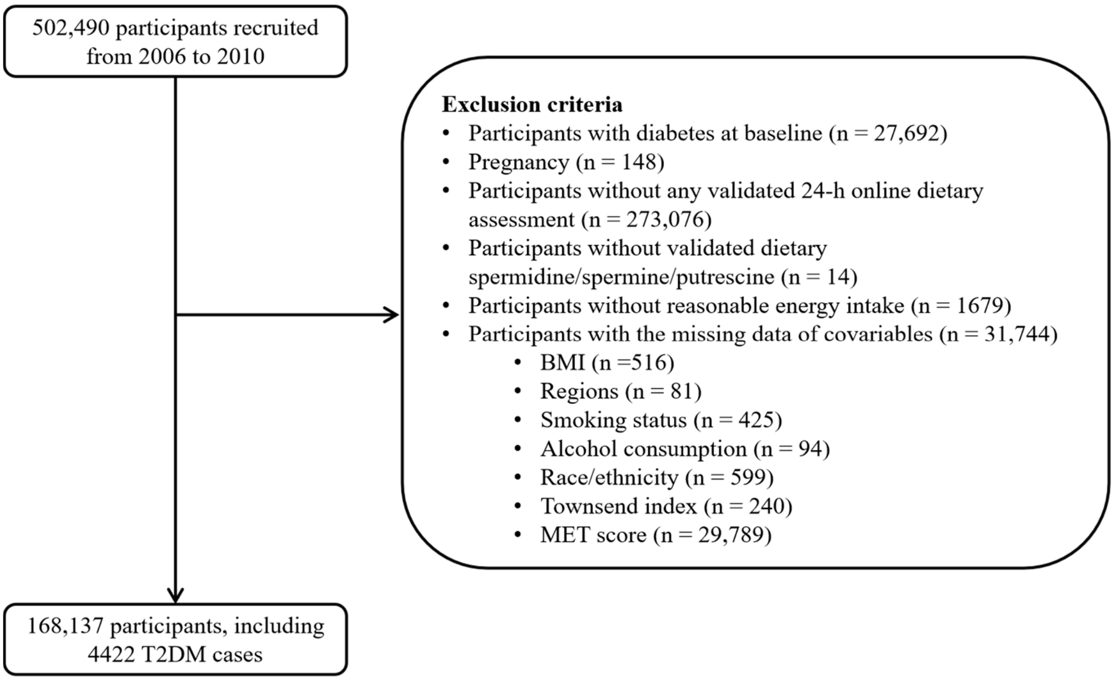

Table S1. Derivation of variables used in analysis from the UK Biobank questionnaire and interviews

Variables	Categories used in analysis	UK Biobank variable used (question ID) and source
**Health outcome**		
Incident T2DM	No; Yes	ICD-10: Diabetes (E11, E12, E13, E14)
**Demographics**		
Sex	Men; Women	Sex (ID: 31) *
Age	Age	Age at recruitment (ID: 21022) *
Race/ethnicity	White; Non-white	Ethnic background (ID: 21000) *
Regions	England; Northern Ireland; Republic of Ireland; Scotland; Wales; Elsewhere	Country of birth (UK/elsewhere) (ID: 1647) *
**Socioeconomic status**		
Townsend index	Quintiles (high index indicates most deprivation)	Townsend index (ID: 189) *
Education	Higher degree (college or university degree, or professional qualifications);<br>Any school degree (A levels, AS levels, O levels, GCSEs or CSEs);<br>Vocational qualifications (NVQ, HND or HNC); Other (none of the above qualifications)	Qualifications (ID: 6138) †
**Behavior risk factors**		
Smoking status	Never; Current, Previous	Smoking status (ID: 20116) †
Alcohol consumption	Never; Current, Previous	Alcohol drinker status (ID: 20117) †
Physical activity (IPAQ MET scores)	Low (<600 metabolic equivalent (MET)-minutes per week);<br>Moderate (≥600 and <3000 metabolic equivalent (MET)-minutes per week);<br>High (≥ 3000 metabolic equivalent (MET)-minutes per week)	Number of days/week of vigorous physical activity 10+ minutes (ID: 904) †;<br>Duration of vigorous activity (ID: 914) †;<br>Number of days/week of moderate physical activity 10+ minutes (ID: 884) †;<br>Duration of moderate activity (ID: 894) †;<br>Number of days/week walked 10+ minutes (ID: 864) †;<br>Duration of walks (ID: 874) †
Sleep duration	Sleep duration	Sleep duration (ID: 1160) †
**Health history/conditions at baseline**		
Family history of diabetes	No; Yes (father or/and mother was/were diagnosed with any types of diabetes)	Father or/and mother was/were diagnosed with any types of diabetes (ID: 20107; 20110) †

To be continued

Table S1-continued

Hypertension (%)	No; Yes (if diagnosed by doctor or taking medication for blood pressure)	Vascular/heart problems diagnosed by doctor (high blood pressure is one response) (ID: 6150) †;<br>Medication for cholesterol, blood pressure or diabetes (men) (ID: 6177) †;<br>Medication for cholesterol, blood pressure, diabetes, or take exogenous hormones (women) (ID: 6153) †;<br>Noncancer illness diagnosed by nurses during verbal interview (ID: 20002) #
Cardiovascular disease (%)	No; Yes (if diagnosed by doctor)	Vascular/heart problems diagnosed by doctor (heart attack, angina, stroke) (ID:6150) †;<br>Noncancer illness diagnosed by nurses during verbal interview (ID: 20002) #
Hyperlipidemia (%)	No; Yes (if diagnosed by doctor or taking cholesterol lowering medication)	Medication for cholesterol, blood pressure or diabetes (men) (ID: 6177) †;<br>Medication for cholesterol, blood pressure, diabetes, or take exogenous hormones (women) (ID: 6153) †;<br>Noncancer illness diagnosed by nurses during verbal interview (ID: 20002) #
Diabetes	No; Yes (if diagnosed by doctor or taking medication for diabetes)	Diabetes diagnosed by doctor (ID: 2443) †;<br>Medication for cholesterol, blood pressure or diabetes (men) (ID: 6177) †;<br>Medication for cholesterol, blood pressure, diabetes, or take exogenous hormones (women) (ID: 6153) †;<br>Noncancer illness diagnosed by nurses during verbal interview (ID: 20002) #
BMI group	Underweight (<18.5); Healthy weight (18.5 to 25);<br>Overweight (25 to 30); Obese (≥30)	BMI (ID: 21001) ‡
Note: *Recruitment questions, †Touchscreen questions, ‡Physical measurements, #Verbal interview		

Table S2. Baseline characteristics of the study population according to quintiles of dietary spermine*

	Overall	Q1 (<2.6 mg/day)	Q2 (2.6~3.5 mg/day)	Q3 (3.5~4.4 mg/day)	Q4 (4.4~5.7 mg/day)	Q5 (>5.7 mg/day)	p-value
n	168,137	33,628	33,627	33,627	33,627	33,628	
Follow-up, years, median [IQR]	12.4 [11.8, 13.2]	12.4[11.8, 13.2]	12.4 [11.9, 13.2]	12.4 [11.8, 13.2]	12.4 [11.8, 13.2]	12.4 [11.8, 13.2]	<0.001
Spermine, mg/day	4.3 ± 2.1	1.9 ± 0.5	3.1 ± 0.2	3.9 ± 0.3	5.0 ± 0.4	7.5 ± 1.9	<0.001
Incident T2DM, n (%)							<0.001
No	163,715 (97.4)	32,806 (97.6)	32,821 (97.6)	32,774 (97.5)	32,714 (97.3)	32,600 (96.9)	
Yes	4422 (2.6)	822 (2.4)	806 (2.4)	853 (2.5)	913 (2.7)	1028 (3.1)	
Age	55.8 ± 8.0	55.4 ± 7.9	55.8 ± 7.9	55.9 ± 7.9	55.9 ± 8.0	55.8 ± 8.2	
Sex, n (%)							<0.001
Women	91,020 (54.1)	20,326 (60.4)	19,545 (58.1)	18,571 (55.2)	17,131 (50.9)	15,447 (45.9)	
Men	77,117 (45.9)	13,302 (39.6)	14,082 (41.9)	15,056 (44.8)	16,496 (49.1)	18,181 (54.1)	
Regions, n (%)							<0.001
England	136,222 (81.0)	26,905 (80.0)	27,468 (81.7)	27,332 (81.3)	27,367 (81.4)	27,150 (80.7)	
Northern Ireland	1146 (0.7)	236 (0.7)	240 (0.7)	212 (0.6)	233 (0.7)	225 (0.7)	
Republic of Ireland	1417 (0.8)	312 (0.9)	269 (0.8)	307 (0.9)	251 (0.7)	278 (0.8)	
Scotland	10,502 (6.2)	1974 (5.9)	2021 (6.0)	2127 (6.3)	2206 (6.6)	2174 (6.5)	
Wales	6198 (3.7)	1129 (3.4)	1259 (3.7)	1287 (3.8)	1231 (3.7)	1292 (3.8)	
Elsewhere	12,652 (7.5)	3072 (9.1)	2370 (7.0)	2362 (7.0)	2339 (7.0)	2509 (7.5)	
Ethnicity, n (%)							<0.001
White race	161,683 (96.2)	31,990 (95.1)	32,536 (96.8)	32,470 (96.6)	32,463 (96.5)	32,224 (95.8)	
Non-white race	6454 (3.8)	1638 (4.9)	1091 (3.2)	1157 (3.4)	1164 (3.5)	1404 (4.2)	
Educational level, n (%)							<0.001
Higher degree	101,032 (60.1)	19,710 (58.6)	20,616 (61.3)	20,549 (61.1)	20,510 (61.0)	19,647 (58.4)	
Any school degree	47,138 (28.0)	9685 (28.8)	9223 (27.4)	9366 (27.9)	9210 (27.4)	9654 (28.7)	
Vocational qualifications	7792 (4.6)	1535 (4.6)	1431 (4.3)	1454 (4.3)	1603 (4.8)	1769 (5.3)	
Other	12,175 (7.2)	2698 (8.0)	2357 (7.0)	2258 (6.7)	2304 (6.9)	2558 (7.6)	
Townsend index, n (%)							<0.001
Q1	33,641 (20.0)	6103 (18.1)	6752 (20.1)	6915 (20.6)	6940 (20.6)	6931 (20.6)	
Q2	33,642 (20.0)	6330 (18.8)	6715 (20.0)	6761 (20.1)	6982 (20.8)	6854 (20.4)	
Q3	33,607 (20.0)	6525 (19.4)	6751 (20.1)	6845 (20.4)	6855 (20.4)	6631 (19.7)	
Q4	33,619 (20.0)	6903 (20.5)	6802 (20.2)	6649 (19.8)	6560 (19.5)	6705 (19.9)	
Q5	33,628 (20.0)	7767 (23.1)	6607 (19.6)	6457 (19.2)	6290 (18.7)	6507 (19.3)	

To be continued

Table S2-continued

Physical activity, n (%)							<0.001
Low	29,989 (17.8)	6288 (18.7)	6061 (18.0)	5833 (17.3)	5860 (17.4)	5947 (17.7)	
Moderate	89,800 (53.4)	17,777 (52.9)	18,128 (53.9)	18,180 (54.1)	18,091 (53.8)	17,624 (52.4)	
High	48,348 (28.8)	9563 (28.4)	9438 (28.1)	9614 (28.6)	9676 (28.8)	10,057 (29.9)	
Smoking status, n (%)							<0.001
Never	95,777 (57.0)	19,170 (57.0)	19,368 (57.6)	19,214 (57.1)	19,066 (56.7)	18,959 (56.4)	
Current	13,002 (7.7)	2941 (8.7)	2602 (7.7)	2399 (7.1)	2506 (7.5)	2554 (7.6)	
Previous	59,358 (35.3)	11,517 (34.2)	11,657 (34.7)	12,014 (35.7)	12,055 (35.8)	12,115 (36.0)	
Alcohol consumption, n (%)							<0.001
Never	4920 (2.9)	1254 (3.7)	970 (2.9)	868 (2.6)	917 (2.7)	911 (2.7)	
Current	158,481 (94.3)	31,181 (92.7)	31,707 (94.3)	31,876 (94.8)	31,928 (94.9)	31,789 (94.5)	
Previous	4736 (2.8)	1193 (3.5)	950 (2.8)	883 (2.6)	782 (2.3)	928 (2.8)	
Sleep duration, hours	7.2 ± 1.0	7.2 ± 1.1	7.2 ± 1.0	7.2 ± 1.0	7.2 ± 1.0	7.1 ± 1.0	<0.001
History of family, n (%)							<0.001
No	134,902 (80.2)	27,053 (80.4)	27,101 (80.6)	26,860 (79.9)	26,918 (80.0)	26,970 (80.2)	
Yes	33,235 (19.8)	6575 (19.6)	6526 (19.4)	6767 (20.1)	6709 (20.0)	6658 (19.8)	
Hypertension, n (%)							<0.001
No	128,474 (76.4)	25,987 (77.3)	25,912 (77.1)	25,792 (76.7)	25,513 (75.9)	25,270 (75.1)	
Yes	39,663 (23.6)	7641 (22.7)	7715 (22.9)	7835 (23.3)	8114 (24.1)	8358 (24.9)	
Cardiovascular disease, n (%)							<0.001
No	161,475 (96.0)	32,388 (96.3)	32,341 (96.2)	32,317 (96.1)	32,280 (96.0)	32,149 (95.6)	
Yes	6662 (4.0)	1240 (3.7)	1286 (3.8)	1310 (3.9)	1347 (4.0)	1479 (4.4)	
Hyperlipidemia, n (%)							<0.001
No	145,054 (86.3)	29,336 (87.2)	29,178 (86.8)	29,063 (86.4)	28,879 (85.9)	28,598 (85.0)	
Yes	23,083 (13.7)	4292 (12.8)	4449 (13.2)	4564 (13.6)	4748 (14.1)	5030 (15.0)	
BMI group[†], n (%)							<0.001
Underweight	935 (0.6)	227 (0.7)	197 (0.6)	182 (0.5)	183 (0.5)	146 (0.4)	
Healthy weight	46,932 (27.9)	10,133 (30.1)	9997 (29.7)	9450 (28.1)	8887 (26.4)	8465 (25.2)	
Overweight	66,137 (39.3)	13,012 (38.7)	13,241 (39.4)	13,311 (39.6)	13,484 (40.1)	13,089 (38.9)	
Obese	54,133 (32.2)	10,256 (30.5)	10,192 (30.3)	10,684 (31.8)	11,073 (32.9)	11,928 (35.5)	

Abbreviations: IQR, interquartile range. Q1- Q5, quintile 1- quintile 5. BMI, body mass index.

[*]Unless otherwise indicated, data are expressed as mean ± SD.

[†]BMI was calculated as weight in kilograms divided by height in meters squared. Underweight: BMI<18.5 kg/m^2; Healthy weight: 18.5≤BMI<24 kg/m^2; Overweight: 24≤BMI<28 kg/m^2; Obese: BMI≥28 kg/m^2.

Table S3. Baseline characteristics of the study population according to quintiles of dietary putrescine*

	Overall	Q1 (<7.2 mg/day)	Q2 (7.2~10.0 mg/day)	Q3 (10.0~13.1 mg/day)	Q4 (13.1~17.6 mg/day)	Q5 (>17.6 mg/day)	p-value
n	168,137	33,628	33,627	33,627	33,627	33,628	
Follow-up, years, median [IQR]	12.4 [11.8, 13.2]	12.4 [11.8, 13.2]	12.5 [11.8, 13.2]	12.4 [11.8, 13.2]	12.4 [11.9, 13.2]	12.4 [11.8, 13.2]	<0.001
Putrescine, mg/day	12.7 ± 6.9	5.0 ± 1.6	8.7 ± 0.8	11.5 ± 0.9	15.1 ± 1.3	23.3 ± 5.9	<0.001
Incident T2DM, n (%)							<0.001
No	163,715 (97.4)	32,602 (96.9)	32,807 (97.6)	32,816 (97.6)	32,820 (97.6)	32,670 (97.2)	
Yes	4422 (2.6)	1026 (3.1)	820 (2.4)	811 (2.4)	807 (2.4)	958 (2.8)	
Age	55.8 ± 8.0	54.6 ± 8.12	55.4 ±8.0	55.9 ± 8.0	56.3 ± 7.8	56.8 ± 7.8	
Sex, n (%)							<0.001
Women	91,020 (54.1)	19,444 (57.8)	18,738 (55.7)	18,220 (54.2)	17,964 (53.4)	16,654 (49.5)	
Men	77,117 (45.9)	14,184 (42.2)	14,889 (44.3)	15,407 (45.8)	15,663 (46.6)	16,974 (50.5)	
Regions, n (%)							<0.001
England	136,222 (81.0)	26,835 (79.8)	27,407 (81.5)	27,587 (82.0)	27,458 (81.7)	26,935 (80.1)	
Northern Ireland	1146 (0.7)	231 (0.7)	213 (0.6)	205 (0.6)	240 (0.7)	257 (0.8)	
Republic of Ireland	1417 (0.8)	278 (0.8)	265 (0.8)	254 (0.8)	262 (0.8)	358 (1.1)	
Scotland	10,502 (6.2)	2115 (6.3)	2125 (6.3)	2112 (6.3)	2065 (6.1)	2,085 (6.2)	
Wales	6198 (3.7)	1160 (3.4)	1221 (3.6)	1209 (3.6)	1325 (3.9)	1283 (3.8)	
Elsewhere	12,652 (7.5)	3009 (8.9)	2396 (7.1)	2260 (6.7)	2277 (6.8)	2710 (8.1)	
Ethnicity, n (%)							<0.001
White race	161,683 (96.2)	31,734 (94.4)	32,452 (96.5)	32,669 (97.2)	32,565 (96.8)	32,263 (95.9)	
Non-white race	6454 (3.8)	1894 (5.6)	1175 (3.5)	958 (2.8)	1062 (3.2)	1365 (4.1)	
Educational level, n (%)							<0.001
Higher degree	101,032 (60.1)	18,224 (54.2)	20,137 (59.9)	20553 (61.1)	21224 (63.1)	20894 (62.1)	
Any school degree	47,138 (28.0)	10,735 (31.9)	9592 (28.5)	9359 (27.8)	8798 (26.2)	8654 (25.7)	
Vocational qualifications	7792 (4.6)	1683 (5.0)	1551 (4.6)	1513 (4.5)	1448 (4.3)	1597 (4.7)	
Other	12,175 (7.2)	2986 (8.9)	2347 (7.0)	2202 (6.5)	2157 (6.4)	2483 (7.4)	
Townsend index, n (%)							<0.001
Q1	33,641 (20.0)	6016 (17.9)	6801 (20.2)	6963 (20.7)	7126 (21.2)	6735 (20.0)	
Q2	33,642 (20.0)	6304 (18.7)	6690 (19.9)	6932 (20.6)	6910 (20.5)	6806 (20.2)	
Q3	33,607 (20.0)	6554 (19.5)	6697 (19.9)	6852 (20.4)	6754 (20.1)	6750 (20.1)	
Q4	33,619 (20.0)	6880 (20.5)	6731 (20.0)	6603 (19.6)	6625 (19.7)	6780 (20.2)	
Q5	33,628 (20.0)	7874 (23.4)	6708 (19.9)	6277 (18.7)	6212 (18.5)	6557 (19.5)	

To be continued

Table S3-continued

Physical activity, n (%)							<0.001
Low	29,989 (17.8)	7282 (21.7)	6589 (19.6)	6090 (18.1)	5349 (15.9)	4679 (13.9)	
Moderate	89,800 (53.4)	17,454 (51.9)	18,079 (53.8)	18,230 (54.2)	18,358 (54.6)	17,679 (52.6)	
High	48,348 (28.8)	8892 (26.4)	8959 (26.6)	9307 (27.7)	9920 (29.5)	11,270 (33.5)	
Smoking status, n (%)							<0.001
Never	95,777 (57.0)	18,924 (56.3)	19,085 (56.8)	19,254 (57.3)	19,427 (57.8)	19,087 (56.8)	
Current	13,002 (7.7)	3527 (10.5)	2719 (8.1)	2409 (7.2)	2152 (6.4)	2195 (6.5)	
Previous	59,358 (35.3)	11,177 (33.2)	11,823 (35.2)	11,964 (35.6)	12,048 (35.8)	12,346 (36.7)	
Alcohol consumption, n (%)							<0.001
Never	4920 (2.9)	1231 (3.7)	923 (2.7)	909 (2.7)	827 (2.5)	1030 (3.1)	
Current	158,481 (94.3)	31,136 (92.6)	31,818 (94.6)	31,892 (94.8)	31,974 (95.1)	31,661 (94.2)	
Previous	4736 (2.8)	1261 (3.7)	886 (2.6)	826 (2.5)	826 (2.5)	937 (2.8)	
Sleep duration, hours	7.2 ± 1.0	7.2 ± 1.1	7.2 ± 1.0	7.2 ± 1.00	7.2 ± 1.0	7.1 ± 1.0	<0.001
History of family, n (%)							<0.001
No	134,902 (80.2)	26,637 (79.2)	26,966 (80.2)	26,982 (80.2)	27,184 (80.8)	27,133 (80.7)	
Yes	33,235 (19.8)	6991 (20.8)	6661 (19.8)	6645 (19.8)	6443 (19.2)	6495 (19.3)	
Hypertension, n (%)							<0.001
No	128,474 (76.4)	26,010 (77.3)	25,924 (77.1)	25,768 (76.6)	25,705 (76.4)	25,067 (74.5)	
Yes	39,663 (23.6)	7618 (22.7)	7703 (22.9)	7859 (23.4)	7922 (23.6)	8561 (25.5)	
Cardiovascular disease, n (%)							<0.001
No	161,475 (96.0)	32,279 (96.0)	32,315 (96.1)	32,382 (96.3)	32,328 (96.1)	32,171 (95.7)	
Yes	6662 (4.0)	1349 (4.0)	1312 (3.9)	1245 (3.7)	1299 (3.9)	1457 (4.3)	
Hyperlipidemia, n (%)							<0.001
No	145,054 (86.3)	29,152 (86.7)	29,113 (86.6)	29,070 (86.4)	29,064 (86.4)	28,655 (85.2)	
Yes	23083 (13.7)	4476 (13.3)	4514 (13.4)	4557 (13.6)	4563 (13.6)	4973 (14.8)	
BMI group†, n (%)							<0.001
Underweight	935 (0.6)	184 (0.5)	194 (0.6)	190 (0.6)	198 (0.6)	169 (0.5)	
Healthy weight	46,932 (27.9)	8992 (26.7)	9526 (28.3)	9576 (28.5)	9631 (28.6)	9207 (27.4)	
Overweight	66,137 (39.3)	12,915 (38.4)	13143 (39.1)	13,409 (39.9)	13,470 (40.1)	13,200 (39.3)	
Obese	54,133 (32.2)	11,537 (34.3)	10,764 (32.0)	10,452 (31.1)	10,328 (30.7)	11,052 (32.9)	

Abbreviations: IQR, interquartile range. Q1- Q5, quintile 1- quintile 5. BMI, body mass index.

*Unless otherwise indicated, data are expressed as mean ± SD.

†BMI was calculated as weight in kilograms divided by height in meters squared. Underweight: BMI<18.5 kg/m^2; Healthy weight: 18.5≤BMI<24 kg/m^2; Overweight: 24≤BMI<28 kg/m^2; Obese: BMI≥28 kg/m^2.

Table S4. The associations of dietary spermine with incident T2DM among subgroups*

	Cases/person-years	Q1 (<2.6 mg/day)	Q2 (2.6~3.5 mg/day)	Q3 (3.5~4.4 mg/day)	Q4 (4.4~5.7 mg/day)	Q5 (>5.7 mg/day)	p for interaction
Age group							<0.001
<60	2,012/1,278,265	1.00	0.86 (0.74, 0.99)	0.97 (0.85, 1.12)	0.90 (0.78, 1.03)	1.02 (0.89, 1.18)	
≥60	2,410/793,482	1.00	1.16 (1.02, 1.33)	1.09 (0.95, 1.25)	1.24 (1.08, 1.42)	1.19 (1.03, 1.36)	
Sex							0.27
Men	2,670/942,905	1.00	1.04 (0.91, 1.19)	1.07 (0.94, 1.22)	1.17 (1.03, 1.33)	1.16 (1.02, 1.32)	
Women	1,752/1,128,842	1.00	0.96 (0.83, 1.11)	0.97 (0.84, 1.13)	0.91 (0.78, 1.07)	1.06 (0.91, 1.24)	
Smoking status							0.26
Never	1,976/1,188,596	1.00	1.00 (0.87, 1.16)	0.96 (0.83, 1.11)	1.01 (0.87, 1.16)	1.06 (0.91, 1.22)	
Current	511/157,194	1.00	0.73 (0.55, 0.96)	0.96 (0.73, 1.26)	0.92 (0.70, 1.22)	1.01 (0.76, 1.35)	
Previous	1,935/725,956	1.00	1.11 (0.95, 1.30)	1.13 (0.97, 1.32)	1.19 (1.03, 1.39)	1.23 (1.05, 1.43)	
Alcohol consumption							0.43
Never	220/59,811	1.00	0.93 (0.62, 1.39)	0.88 (0.58, 1.34)	0.77 (0.50, 1.18)	0.95 (0.62, 1.45)	
Current	3,962/1,955,145	1.00	1.01 (0.91, 1.13)	1.02 (0.92, 1.13)	1.09 (0.98, 1.21)	1.12 (1.01, 1.24)	
Previous	240/56,791	1.00	1.09 (0.72, 1.65)	1.36 (0.92, 2.02)	1.05 (0.67, 1.64)	1.38 (0.91, 2.08)	
Townsend index							0.66
Q1	670/420,573	1.00	1.14 (0.87, 1.48)	1.16 (0.90, 1.51)	1.20 (0.92, 1.56)	1.14 (0.87, 1.49)	
Q2	807/416,208	1.00	1.06 (0.89, 1.27)	1.07 (0.90, 1.28)	1.16 (0.97, 1.38)	1.14 (0.95, 1.36)	
Q3	849/414,799	1.00	1.08 (0.86, 1.37)	1.13 (0.90, 1.42)	1.24 (0.98, 1.55)	1.35 (1.07, 1.70)	
Q4	897/411,704	1.00	1.05 (0.86, 1.30)	1.06 (0.85, 1.31)	0.96 (0.77, 1.20)	1.02 (0.82, 1.27)	
Q5	1,199/408,462	1.00	0.87 (0.73, 1.05)	0.92 (0.77, 1.10)	0.95 (0.79, 1.14)	1.03 (0.85, 1.23)	
Race/ethnicity							0.04
White	4,101/1,994,658	1.00	1.00 (0.90, 1.11)	1.01 (0.91, 1.12)	1.08 (0.98, 1.20)	1.10 (0.99, 1.22)	
Non-white	321/77,089	1.00	1.10 (0.77, 1.58)	1.28 (0.91, 1.78)	0.81 (0.55, 1.19)	1.32 (0.93, 1.86)	
Physical activity							0.25
Low	1,118/367,879	1.00	0.96 (0.80, 1.16)	0.95 (0.79, 1.15)	0.99 (0.82, 1.20)	0.89 (0.73, 1.08)	
Moderate	2,208/1,109,518	1.00	1.02 (0.89, 1.18)	0.99 (0.86, 1.14)	1.09 (0.95, 1.25)	1.15 (1.00, 1.32)	
High	1,096/594,350	1.00	1.02 (0.83, 1.25)	1.20 (0.99, 1.46)	1.11 (0.91, 1.36)	1.30 (1.06, 1.59)	
Educational level							0.16
Higher degree	2,166/1,252,991	1.00	0.93 (0.80, 1.07)	1.03 (0.90, 1.18)	1.03 (0.89, 1.18)	1.05 (0.91, 1.21)	
Any school degree	1,280/579,043	1.00	1.10 (0.91, 1.32)	1.07 (0.89, 1.29)	1.12 (0.93, 1.34)	1.14 (0.94, 1.37)	
Vocational qualifications	314/145,227	1.00	1.09 (0.74, 1.62)	1.28 (0.88, 1.88)	1.36 (0.93, 1.99)	1.41 (0.96, 2.07)	
Other	662/94,487	1.00	1.01 (0.79, 1.28)	0.76 (0.59, 0.99)	0.89 (0.69, 1.14)	1.05 (0.83, 1.34)	

To be continued

Table S4-continued

	Cases/person-years	Q1 (<2.6 mg/day)	Q2 (2.6~3.5 mg/day)	Q3 (3.5~4.4 mg/day)	Q4 (4.4~5.7 mg/day)	Q5 (>5.7 mg/day)	p for interaction
Family history of diabetes							0.58
No	2,932/1,666,274	1.00	1.05 (0.93, 1.18)	1.06 (0.94, 1.19)	1.10 (0.97, 1.24)	1.10 (0.97, 1.25)	
Yes	1,490/405,473	1.00	0.93 (0.78, 1.10)	0.97 (0.82, 1.15)	1.01 (0.85, 1.19)	1.13 (0.96, 1.34)	
Hypertension							0.84
No	2,203/159,4562	1.00	0.98 (0.86, 1.13)	1.06 (0.92, 1.22)	1.07 (0.93, 1.22)	1.14 (1.00, 1.32)	
Yes	2,219/477,185	1.00	1.03 (0.90, 1.18)	1.00 (0.87, 1.15)	1.06 (0.93, 1.22)	1.08 (0.94, 1.24)	
Cardiovascular disease							0.93
No	3,868/1,993,952	1.00	1.02 (0.92, 1.13)	1.04 (0.94, 1.15)	1.07 (0.96, 1.19)	1.12 (1.01, 1.25)	
Yes	554/77,795	1.00	0.92 (0.70, 1.22)	0.93 (0.70, 1.23)	1.05 (0.79, 1.38)	1.04 (0.79, 1.38)	
Hyperlipidemia							0.19
No	2,930/1,797,116	1.00	1.07 (0.95, 1.20)	1.05 (0.93, 1.18)	1.05 (0.93, 1.19)	1.16 (1.03, 1.31)	
Yes	1,492/274,631	1.00	0.90 (0.76, 1.07)	0.99 (0.84, 1.17)	1.08 (0.92, 1.28)	1.02 (0.86, 1.21)	
BMI group†							0.30
Underweight/ healthy weight	338/597,623	1.00	0.93 (0.68, 1.29)	0.76 (0.53, 1.08)	0.97 (0.69, 1.36)	0.92 (0.64, 1.31)	
Overweight	1,086/819,249	1.00	1.06 (0.88, 1.28)	1.22 (1.01, 1.47)	1.13 (0.94, 1.36)	1.30 (1.08, 1.56)	
Obese	2,998/654,876	1.00	1.02 (0.90, 1.15)	1.02 (0.91, 1.15)	1.02 (0.91, 1.15)	1.07 (0.95, 1.21)	

*Models was adjusted for age, sex, race/ethnicity, regions, educational level, Townsend index, smoking status, drinking status, physical activity, sleep duration and total energy intake, family history of diabetes, hypertension at baseline, cardiovascular disease at baseline, hyperlipidemia at baseline and BMI group.

†BMI was calculated as weight in kilograms divided by height in meters squared. Underweight: BMI<18.5 kg/m^2; Healthy weight: 18.5≤BMI<24 kg/m^2; Overweight: 24≤BMI<28 kg/m^2; Obese: BMI≥28 kg/m^2.

Table S5. The associations of dietary putrescine with incident T2DM among subgroups*

	Cases/person-years	Q1 (<7.2 mg/day)	Q2 (7.2~10.0 mg/day)	Q3 (10.0~13.1 mg/day)	Q4 (13.1~17.6 mg/day)	Q5 (>17.6 mg/day)	p for interaction
Age group							0.51
<60	2,012/1,278,265	1.00	0.87 (0.76, 0.99)	0.89 (0.78, 1.02)	0.81 (0.70, 0.93)	0.92 (0.80, 1.05)	
≥60	2,410/793,482	1.00	0.83 (0.73, 0.94)	0.81 (0.71, 0.92)	0.85 (0.74, 0.96)	0.89 (0.78, 1.01)	
Sex							0.46
Men	2,670/942,905	1.00	0.83 (0.73, 0.93)	0.84 (0.75, 0.95)	0.87 (0.77, 0.98)	0.94 (0.83, 1.06)	
Women	1,752/1,128,842	1.00	0.85 (0.74, 0.98)	0.81 (0.70, 0.94)	0.74 (0.64, 0.86)	0.78 (0.67, 0.91)	
Smoking status							0.68
Never	1,976/1,188,596	1.00	0.83 (0.72, 0.95)	0.78 (0.68, 0.90)	0.82 (0.71, 0.95)	0.88 (0.76, 1.01)	
Current	511/157,194	1.00	0.94 (0.73, 1.22)	0.82 (0.62, 1.08)	0.82 (0.61, 1.10)	1.05 (0.80, 1.39)	
Previous	1,935/725,956	1.00	0.82 (0.71, 0.94)	0.88 (0.76, 1.01)	0.81 (0.70, 0.94)	0.84 (0.73, 0.97)	
Alcohol consumption							0.85
Never	220/59,811	1.00	0.80 (0.54, 1.18)	0.80 (0.52, 1.22)	0.76 (0.49, 1.18)	0.81 (0.54, 1.22)	
Current	3,962/1,955,145	1.00	0.84 (0.76, 0.93)	0.84 (0.76, 0.93)	0.82 (0.74, 0.91)	0.87 (0.79, 0.96)	
Previous	240/56,791	1.00	0.81 (0.54, 1.20)	0.69 (0.45, 1.06)	0.83 (0.55, 1.24)	1.12 (0.77, 1.62)	
Townsend index							0.03
Q1	670/420,573	1.00	0.85 (0.66, 1.09)	0.84 (0.65, 1.08)	1.11 (0.87, 1.41)	0.94 (0.73, 1.21)	
Q2	807/416,208	1.00	0.86 (0.73, 1.02)	0.91 (0.77, 1.07)	0.96 (0.82, 1.14)	0.93 (0.79, 1.10)	
Q3	849/414,799	1.00	0.97 (0.78, 1.20)	0.87 (0.70, 1.09)	0.94 (0.76, 1.18)	1.00 (0.81, 1.25)	
Q4	897/411,704	1.00	0.78 (0.64, 0.95)	0.68 (0.55, 0.84)	0.65 (0.53, 0.81)	0.75 (0.61, 0.92)	
Q5	1,199/408,462	1.00	0.80 (0.67, 0.95)	0.87 (0.72, 1.03)	0.72 (0.59, 0.86)	0.86 (0.72, 1.02)	
Race/ethnicity							0.50
White	4,101/1,994,658	1.00	0.82 (0.74, 0.90)	0.83 (0.75, 0.91)	0.81 (0.73, 0.89)	0.87 (0.79, 0.96)	
Non-white	321/77,089	1.00	1.11 (0.81, 1.54)	0.92 (0.64, 1.32)	0.90 (0.63, 1.28)	0.89 (0.64, 1.25)	
Physical activity							0.06
Low	1,118/367,879	1.00	0.82 (0.69, 0.97)	0.67 (0.56, 0.81)	0.69 (0.57, 0.83)	0.86 (0.71, 1.03)	
Moderate	2,208/1,109,518	1.00	0.84 (0.73, 0.96)	0.93 (0.82, 1.06)	0.84 (0.74, 0.97)	0.93 (0.81, 1.06)	
High	1,096/594,350	1.00	0.87 (0.71, 1.05)	0.83 (0.68, 1.01)	0.91 (0.75, 1.10)	0.83 (0.68, 1.00)	
Educational level							0.14
Higher degree	2,166/1,252,991	1.00	0.83 (0.73, 0.95)	0.79 (0.68, 0.90)	0.81 (0.70, 0.92)	0.82 (0.71, 0.93)	
Any school degree	1,280/579,043	1.00	0.89 (0.75, 1.06)	0.96 (0.80, 1.14)	0.91 (0.76, 1.09)	1.06 (0.89, 1.26)	
Vocational qualifications	314/145,227	1.00	0.79 (0.55, 1.12)	0.93 (0.65, 1.32)	0.89 (0.62, 1.29)	1.00 (0.71, 1.43)	
Other	662/94,487	1.00	0.76 (0.61, 0.96)	0.69 (0.54, 0.88)	0.63 (0.49, 0.81)	0.71 (0.56, 0.90)	

To be continued

Table S5-continued

	Cases/person-years	Q1 (<7.2 mg/day)	Q2 (7.2~10.0 mg/day)	Q3 (10.0~13.1 mg/day)	Q4 (13.1~17.6 mg/day)	Q5 (>17.6 mg/day)	p for interaction
Family history of diabetes							0.07
No	2,932/1,666,274	1.00	0.81 (0.72, 0.91)	0.88 (0.79, 0.99)	0.81 (0.72, 0.92)	0.85 (0.76, 0.96)	
Yes	1,490/405,473	1.00	0.89 (0.76, 1.04)	0.73 (0.62, 0.86)	0.82 (0.69, 0.96)	0.92 (0.78, 1.07)	
Hypertension							0.20
No	2,203/159,4562	1.00	0.84 (0.74, 0.96)	0.92 (0.81, 1.05)	0.85 (0.74, 0.97)	0.91 (0.80, 1.04)	
Yes	2,219/477,185	1.00	0.83 (0.73, 0.95)	0.75 (0.65, 0.86)	0.79 (0.69, 0.90)	0.84 (0.74, 0.96)	
Cardiovascular disease							0.19
No	3,868/1,993,952	1.00	0.82 (0.74, 0.90)	0.84 (0.76, 0.93)	0.81 (0.73, 0.90)	0.86 (0.77, 0.95)	
Yes	554/77,795	1.00	0.94 (0.73, 1.22)	0.71 (0.53, 0.95)	0.86 (0.66, 1.13)	1.03 (0.79, 1.33)	
Hyperlipidemia							0.80
No	2,930/1,797,116	1.00	0.81 (0.72, 0.91)	0.83 (0.74, 0.93)	0.80 (0.72, 0.90)	0.84 (0.75, 0.95)	
Yes	1,492/274,631	1.00	0.90 (0.77, 1.06)	0.84 (0.71, 0.99)	0.85 (0.72, 1.01)	0.96 (0.82, 1.13)	
BMI group†							0.21
Underweight/healthy	338/597,623	1.00	1.06 (0.76, 1.48)	0.88 (0.61, 1.25)	0.88 (0.61, 1.27)	1.13 (0.80, 1.59)	
Overweight	1,086/819,249	1.00	0.72 (0.60, 0.88)	0.81 (0.67, 0.97)	0.75 (0.62, 0.91)	0.81 (0.67, 0.97)	
Obese	2,998/654,876	1.00	0.86 (0.77, 0.96)	0.84 (0.75, 0.94)	0.84 (0.74, 0.94)	0.88 (0.79, 0.99)	

*Models was adjusted for age, sex, race/ethnicity, regions, educational level, Townsend index, smoking status, drinking status, physical activity, sleep duration and total energy intake, family history of diabetes, hypertension at baseline, cardiovascular disease at baseline, hyperlipidemia at baseline and BMI group.

†BMI was calculated as weight in kilograms divided by height in meters squared. Underweight: BMI<18.5 kg/m^2; Healthy weight: 18.5≤BMI<24 kg/m^2; Overweight: 24≤BMI<28 kg/m^2; Obese: BMI≥28 kg/m^2.

Figure S2. The associations of dietary polyamine with incident T2DM among participants after imputing missing values of covariables

A

Spermidine	Cases/person-years	HR (95%CI)	*p* value
**Model 1**			
Q1	1248/487,692	1.00 (ref)	
Q2	987/493,757	0.77 (0.71, 0.84)	<0.001
Q3	999/494,143	0.76 (0.70, 0.83)	<0.001
Q4	1046/493,160	0.78 (0.72, 0.85)	<0.001
Q5	1245/490,351	0.91 (0.84, 0.98)	0.01
**Model 2**			
Q1	1248/487,692	1.00 (ref)	
Q2	987/493,757	0.82 (0.76, 0.89)	<0.001
Q3	999/494,143	0.83 (0.76, 0.90)	<0.001
Q4	1046/493,160	0.84 (0.78, 0.92)	<0.001
Q5	1245/490,351	0.95 (0.88, 1.03)	0.22
**Model 3**			
Q1	1248/487,692	1.00 (ref)	
Q2	987/493,757	0.84 (0.77, 0.91)	<0.001
Q3	999/494,143	0.85 (0.78, 0.93)	<0.001
Q4	1046/493,160	0.88 (0.80, 0.95)	0.003
Q5	1245/490,351	0.99 (0.90, 1.07)	0.74
**Model 4**			
Q1	1248/487,692	1.00 (ref)	
Q2	987/493,757	0.84 (0.77, 0.92)	<0.001
Q3	999/494,143	0.85 (0.78, 0.93)	<0.001
Q4	1046/493,160	0.88 (0.80, 0.95)	0.003
Q5	1245/490,351	0.95 (0.87, 1.04)	0.28
**Model 5**			
Q1	1248/487,692	1.00 (ref)	
Q2	987/493,757	0.87 (0.80, 0.95)	0.001
Q3	999/494,143	0.88 (0.81, 0.96)	0.003
Q4	1046/493,160	0.91 (0.84, 0.99)	0.03
Q5	1245/490,351	0.96 (0.88, 1.04)	0.33

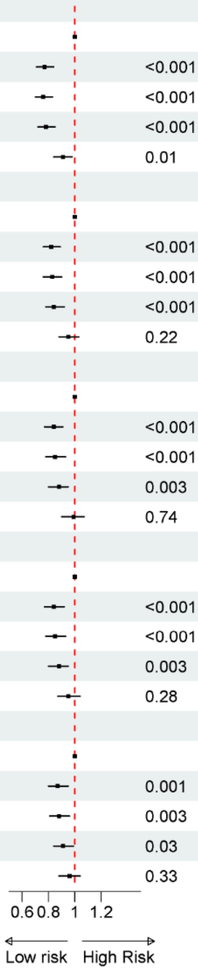

B

Spermine	Cases/person-years	HR (95%CI)	*p* value
**Model 1**			
Q1	1035/490,102	1.00 (ref)	
Q2	1011/493,027	0.96 (0.88, 1.05)	0.36
Q3	1068/492,955	0.99 (0.91, 1.08)	0.9
Q4	1138/492,424	1.04 (0.96, 1.13)	0.34
Q5	1273/490,595	1.13 (1.04, 1.23)	0.003
**Model 2**			
Q1	1035/490,102	1.00 (ref)	
Q2	1011/493,027	1.00 (0.92, 1.09)	1
Q3	1068/492,955	1.04 (0.95, 1.13)	0.38
Q4	1138/492,424	1.09 (1.00, 1.18)	0.05
Q5	1273/490,595	1.17 (1.07, 1.27)	<0.001
**Model 3**			
Q1	1035/490,102	1.00 (ref)	
Q2	1011/493,027	1.02 (0.94, 1.12)	0.60
Q3	1068/492,955	1.07 (0.99, 1.17)	0.10
Q4	1138/492,424	1.13 (1.03, 1.23)	0.008
Q5	1273/490,595	1.21 (1.11, 1.32)	<0.001
**Model 4**			
Q1	1035/490,102	1.00 (ref)	
Q2	1011/493,027	1.01 (0.93, 1.10)	0.81
Q3	1068/492,955	1.05 (0.96, 1.14)	0.30
Q4	1138/492,424	1.09 (1.00, 1.19)	0.05
Q5	1273/490,595	1.16 (1.07, 1.27)	<0.001
**Model 5**			
Q1	1035/490,102	1.00 (ref)	
Q2	1011/493,027	1.01 (0.92, 1.10)	0.86
Q3	1068/492,955	1.03 (0.94, 1.12)	0.53
Q4	1138/492,424	1.06 (0.97, 1.16)	0.20
Q5	1273/490,595	1.10 (1.01, 1.20)	0.04

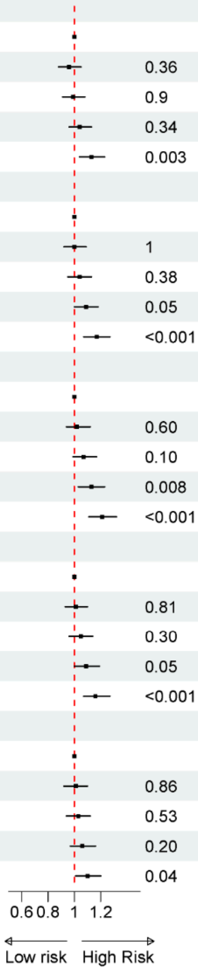

C

Putrescine	Cases/person-years	HR (95%CI)	*p* value
**Model 1**			
Q1	1264/488,795	1.00 (ref)	
Q2	1014/492,973	0.78 (0.71, 0.84)	<0.001
Q3	1024/492,933	0.76 (0.70, 0.83)	<0.001
Q4	1030/492,999	0.75 (0.69, 0.82)	<0.001
Q5	1193/491,403	0.83 (0.77, 0.90)	<0.001
**Model 2**			
Q1	1264/488,795	1.00 (ref)	
Q2	1014/492,973	0.82 (0.75, 0.89)	<0.001
Q3	1024/492,933	0.82 (0.76, 0.89)	<0.001
Q4	1030/492,999	0.82 (0.75, 0.89)	<0.001
Q5	1193/491,403	0.88 (0.82, 0.96)	0.002
**Model 3**			
Q1	1264/488,795	1.00 (ref)	
Q2	1014/492,973	0.83 (0.77, 0.91)	<0.001
Q3	1024/492,933	0.84 (0.77, 0.92)	<0.001
Q4	1030/492,999	0.85 (0.78, 0.92)	<0.001
Q5	1193/491,403	0.91 (0.84, 0.99)	0.04
**Model 4**			
Q1	1264/488,795	1.00 (ref)	
Q2	1014/492,973	0.83 (0.77, 0.91)	<0.001
Q3	1024/492,933	0.84 (0.77, 0.91)	<0.001
Q4	1030/492,999	0.84 (0.78, 0.92)	<0.001
Q5	1193/491,403	0.90 (0.83, 0.98)	0.01
**Model 5**			
Q1	1264/488,795	1.00 (ref)	
Q2	1014/492,973	0.85 (0.78, 0.92)	<0.001
Q3	1024/492,933	0.86 (0.79, 0.94)	<0.001
Q4	1030/492,999	0.86 (0.79, 0.94)	<0.001
Q5	1193/491,403	0.90 (0.83, 0.98)	0.02

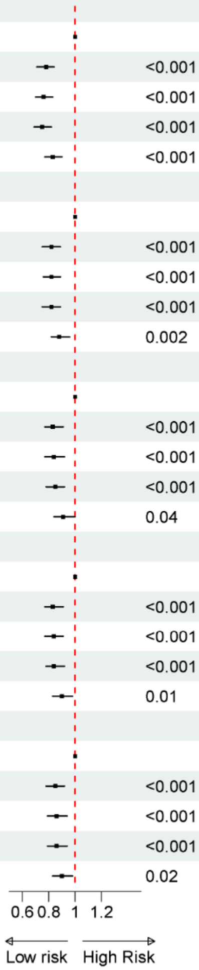

A. The association between dietary spermidine and incident T2DM after imputing physical activity. B. The association between dietary spermine and incident T2DM after imputing physical activity. C. The association between dietary putrescine and incident T2DM after imputing physical activity. Model 1 was adjusted for sociodemographic factors, including age, sex, race/ethnicity, regions. Model 2 was further adjusted for educational level, Townsend index. Model 3 was further adjusted for smoking status, drinking status, physical activity, sleep duration and total energy intake. Model 4 was further adjusted for family history of diabetes, hypertension at baseline, cardiovascular disease at baseline, hyperlipidemia at baseline. Model 5 was further adjusted for BMI group. Abbreviations: HR, hazard ratio. Q1- Q5, quintile 1- quintile 5. BMI, body mass index.

Figure S3. The nonlinear associations of dietary polyamine with incident T2DM among participants after imputing missing values of covariables

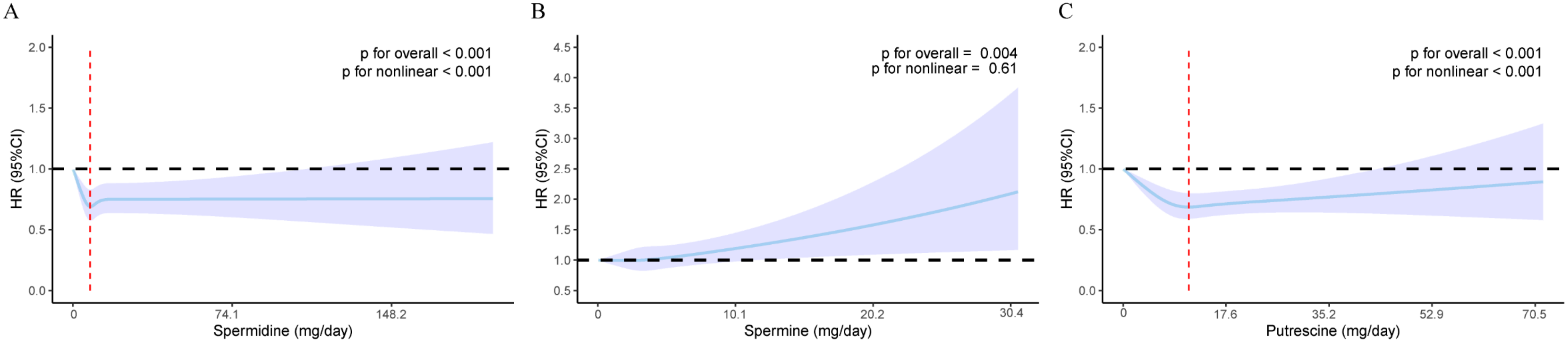

A. The nonlinear association between dietary spermidine and incident T2DM after imputing physical activity. B. The nonlinear association between dietary spermine and incident T2DM after imputing physical activity. C. The nonlinear association between dietary putrescine and incident T2DM after imputing physical activity. Hazard ratio was indicated by solid lines and 95% CIs by shaded areas. The red dashed line indicates the value of dietary spermidine and putrescine at the inflection points. Models were adjusted for age, sex, race/ethnicity, regions, educational level, Townsend index, smoking status, drinking status, physical activity, sleep duration and total energy intake, family history of diabetes, hypertension at baseline, cardiovascular disease at baseline, hyperlipidemia at baseline and BMI group.

Abbreviations: T2DM, type 2 diabetes mellitus. HR, hazard ratio. CI, confidence interval. BMI, body mass index.

Figure S4. The associations of dietary polyamine with incident T2DM after deleting extreme value of dietary polyamine

A

Spermidine	Cases/person-years	HR (95%CI)	*p* value
**Model 1**			
Q1	836/371,479	1.00 (ref)	
Q2	705/374,644	0.83 (0.75, 0.92)	<0.001
Q3	722/374,654	0.83 (0.75, 0.91)	<0.001
Q4	751/373,770	0.84 (0.76, 0.93)	<0.001
Q5	841/372,552	0.91 (0.83, 1.00)	0.06
**Model 2**			
Q1	836/371,479	1.00 (ref)	
Q2	705/374,644	0.86 (0.78, 0.95)	0.003
Q3	722/374,654	0.87 (0.78, 0.96)	0.005
Q4	751/373,770	0.88 (0.80, 0.98)	0.01
Q5	841/372,552	0.94 (0.86, 1.04)	0.24
**Model 3**			
Q1	836/371,479	1.00 (ref)	
Q2	705/374,644	0.87 (0.79, 0.97)	0.008
Q3	722/374,654	0.89 (0.81, 0.99)	0.03
Q4	751/373,770	0.92 (0.83, 1.01)	0.09
Q5	841/372,552	0.98 (0.89, 1.09)	0.76
**Model 4**			
Q1	836/371,479	1.00 (ref)	
Q2	705/374,644	0.87 (0.79, 0.97)	0.009
Q3	722/374,654	0.89 (0.80, 0.98)	0.02
Q4	751/373,770	0.92 (0.83, 1.01)	0.09
Q5	841/372,552	0.95 (0.86, 1.06)	0.36
**Model 5**			
Q1	836/371,479	1.00 (ref)	
Q2	705/374,644	0.89 (0.80, 0.98)	0.02
Q3	722/374,654	0.91 (0.82, 1.00)	0.06
Q4	751/373,770	0.94 (0.85, 1.04)	0.20
Q5	841/372,552	0.95 (0.86, 1.05)	0.34

0.6 0.8 1 1.2

Low risk High Risk

B

Spermine	Cases/person-years	HR (95%CI)	*p* value
**Model 1**			
Q1	686/372,991	1.00 (ref)	
Q2	715/374,068	1.02 (0.92, 1.13)	0.69
Q3	764/374,005	1.07 (0.97, 1.19)	0.19
Q4	799/373,481	1.10 (0.99, 1.22)	0.06
Q5	891/372,554	1.20 (1.09, 1.33)	<0.001
**Model 2**			
Q1	686/372,991	1.00 (ref)	
Q2	715/374,068	1.04 (0.94, 1.16)	0.41
Q3	764/374,005	1.10 (0.99, 1.22)	0.07
Q4	799/373,481	1.13 (1.02, 1.25)	0.02
Q5	891/372,554	1.22 (1.11, 1.35)	<0.001
**Model 3**			
Q1	686/372,991	1.00 (ref)	
Q2	715/374,068	1.06 (0.95, 1.17)	0.31
Q3	764/374,005	1.13 (1.01, 1.25)	0.03
Q4	799/373,481	1.16 (1.04, 1.29)	0.006
Q5	891/372,554	1.26 (1.13, 1.40)	<0.001
**Model 4**			
Q1	686/372,991	1.00 (ref)	
Q2	715/374,068	1.03 (0.93, 1.15)	0.53
Q3	764/374,005	1.09 (0.99, 1.21)	0.09
Q4	799/373,481	1.12 (1.01, 1.25)	0.03
Q5	891/372,554	1.21 (1.09, 1.34)	<0.001
**Model 5**			
Q1	686/372,991	1.00 (ref)	
Q2	715/374,068	1.02 (0.92, 1.13)	0.70
Q3	764/374,005	1.06 (0.96, 1.18)	0.26
Q4	799/373,481	1.07 (0.97, 1.19)	0.18
Q5	891/372,554	1.14 (1.02, 1.26)	0.02

0.6 0.8 1 1.2

Low risk High Risk

C

Putrescine	Cases/person-years	HR (95%CI)	*p* value
**Model 1**			
Q1	877/371,975	1.00 (ref)	
Q2	727/373,929	0.80 (0.73, 0.89)	<0.001
Q3	716/374,116	0.77 (0.70, 0.85)	<0.001
Q4	718/373,921	0.76 (0.68, 0.83)	<0.001
Q5	817/373,158	0.82 (0.74, 0.90)	<0.001
**Model 2**			
Q1	877/371,975	1.00 (ref)	
Q2	727/373,929	0.83 (0.75, 0.92)	<0.001
Q3	716/374,116	0.81 (0.73, 0.89)	<0.001
Q4	718/373,921	0.80 (0.73, 0.89)	<0.001
Q5	817/373,158	0.86 (0.78, 0.94)	0.002
**Model 3**			
Q1	877/371,975	1.00 (ref)	
Q2	727/373,929	0.85 (0.77, 0.94)	0.001
Q3	716/374,116	0.83 (0.75, 0.92)	<0.001
Q4	718/373,921	0.83 (0.75, 0.92)	<0.001
Q5	817/373,158	0.90 (0.82, 0.99)	0.04
**Model 4**			
Q1	877/371,975	1.00 (ref)	
Q2	727/373,929	0.84 (0.76, 0.93)	<0.001
Q3	716/374,116	0.82 (0.74, 0.91)	<0.001
Q4	718/373,921	0.83 (0.75, 0.91)	<0.001
Q5	817/373,158	0.88 (0.80, 0.97)	0.01
**Model 5**			
Q1	877/371,975	1.00 (ref)	
Q2	727/373,929	0.85 (0.77, 0.94)	0.002
Q3	716/374,116	0.84 (0.76, 0.93)	<0.001
Q4	718/373,921	0.84 (0.76, 0.93)	<0.001
Q5	817/373,158	0.88 (0.80, 0.97)	0.01

0.6 0.8 1 1.2

Low risk High Risk

A. The association between dietary spermidine and incident T2DM after deleting extreme value of dietary polyamine. B. The association between dietary spermine and incident T2DM after deleting extreme value of dietary polyamine. C. The association between dietary putrescine and incident T2DM after deleting extreme value of dietary polyamine. Model 1 was adjusted for sociodemographic factors, including age, sex, race/ethnicity, regions. Model 2 was further adjusted for educational level, Townsend index. Model 3 was further adjusted for smoking status, drinking status, physical activity, sleep duration and total energy intake. Model 4 was further adjusted for family history of diabetes, hypertension at baseline, cardiovascular disease at baseline, hyperlipidemia at baseline. Model 5 was further adjusted for BMI group. Abbreviations: HR, hazard ratio. Q1- Q5, quintile 1- quintile 5. BMI, body mass index.

Figure S5. The nonlinear associations of dietary polyamine with incident T2DM after deleting extreme value of dietary polyamine

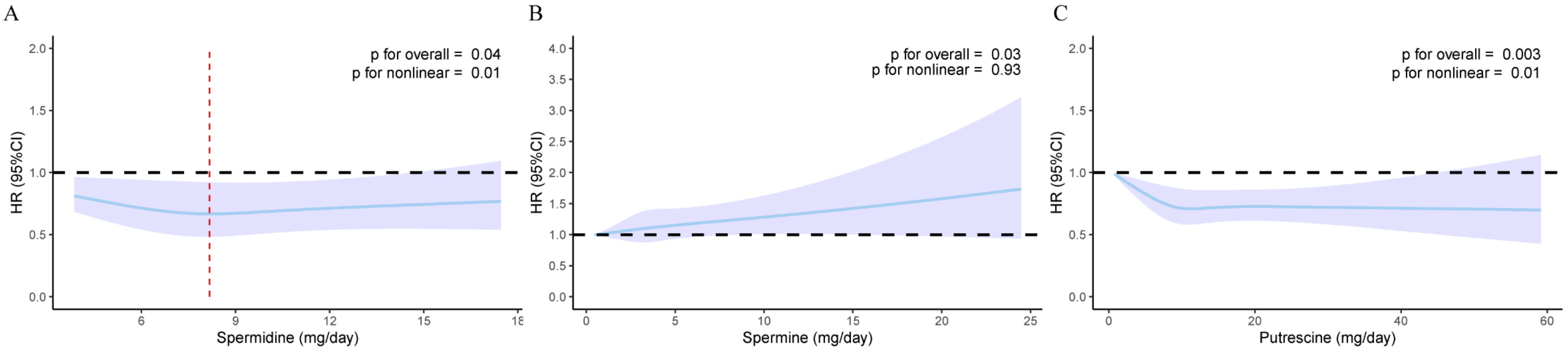

A. The nonlinear association between dietary spermidine and incident T2DM after deleting extreme value of dietary polyamine. B. The nonlinear association between dietary spermine and incident T2DM after deleting extreme value of dietary polyamine. C. The nonlinear association between dietary putrescine and incident T2DM after deleting extreme value of dietary polyamine. Hazard ratio was indicated by solid lines and 95% CIs by shaded areas. The red dashed line indicates the value of dietary spermidine and putrescine at the inflection points. Models were adjusted for age, sex, race/ethnicity, regions, educational level, Townsend index, smoking status, drinking status, physical activity, sleep duration and total energy intake, family history of diabetes, hypertension at baseline, cardiovascular disease at baseline, hyperlipidemia at baseline and BMI group.

Abbreviations: T2DM, type 2 diabetes mellitus. HR, hazard ratio. CI, confidence interval. BMI, body mass index

Figure S6. The associations of dietary polyamine with incident T2DM among participants with two or more diet assessments

A

Spermidine	Cases/person-years	HR (95%CI)	*p* value
**Model 1**			
Q1	555/255,880	1.00 (ref)	
Q2	426/257,575	0.74 (0.65, 0.84)	<0.001
Q3	473/257,340	0.80 (0.70, 0.90)	<0.001
Q4	463/256,742	0.76 (0.67, 0.86)	<0.001
Q5	577/256,000	0.93 (0.83, 1.04)	0.21
**Model 2**			
Q1	555/255,880	1.00 (ref)	
Q2	426/257,575	0.77 (0.68, 0.87)	<0.001
Q3	473/257,340	0.84 (0.74, 0.95)	0.005
Q4	463/256,742	0.80 (0.71, 0.91)	<0.001
Q5	577/256,000	0.95 (0.85, 1.07)	0.40
**Model 3**			
Q1	555/255,880	1.00 (ref)	
Q2	426/257,575	0.79 (0.70, 0.90)	<0.001
Q3	473/257,340	0.88 (0.78, 0.99)	0.04
Q4	463/256,742	0.84 (0.74, 0.96)	0.009
Q5	577/256,000	1.01 (0.89, 1.15)	0.86
**Model 4**			
Q1	555/255,880	1.00 (ref)	
Q2	426/257,575	0.80 (0.70, 0.91)	<0.001
Q3	473/257,340	0.88 (0.77, 0.99)	0.04
Q4	463/256,742	0.84 (0.74, 0.95)	0.008
Q5	577/256,000	0.97 (0.85, 1.10)	0.60
**Model 5**			
Q1	555/255,880	1.00 (ref)	
Q2	426/257,575	0.82 (0.72, 0.93)	0.002
Q3	473/257,340	0.90 (0.79, 1.02)	0.10
Q4	463/256,742	0.87 (0.76, 0.99)	0.03
Q5	577/256,000	0.96 (0.84, 1.09)	0.53

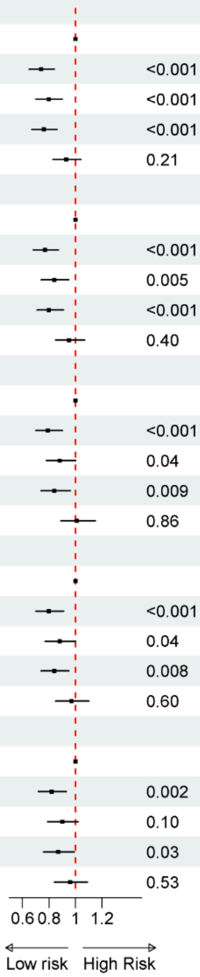

B

Spermine	Cases/person-years	HR (95%CI)	*p* value
**Model 1**			
Q1	449/256,844	1.00 (ref)	
Q2	458/257,043	0.98 (0.86, 1.11)	0.75
Q3	470/256,983	0.98 (0.86, 1.11)	0.74
Q4	540/256,429	1.10 (0.97, 1.24)	0.14
Q5	577/256,237	1.14 (1.00, 1.29)	0.04
**Model 2**			
Q1	449/256,844	1.00 (ref)	
Q2	458/257,043	1.00 (0.88, 1.14)	0.96
Q3	470/256,983	1.00 (0.88, 1.13)	0.96
Q4	540/256,429	1.12 (0.99, 1.27)	0.08
Q5	577/256,237	1.15 (1.02, 1.31)	0.02
**Model 3**			
Q1	449/256,844	1.00 (ref)	
Q2	458/257,043	1.03 (0.90, 1.17)	0.67
Q3	470/256,983	1.03 (0.91, 1.18)	0.63
Q4	540/256,429	1.17 (1.02, 1.33)	0.02
Q5	577/256,237	1.21 (1.06, 1.38)	0.005
**Model 4**			
Q1	449/256,844	1.00 (ref)	
Q2	458/257,043	1.01 (0.89, 1.15)	0.89
Q3	470/256,983	1.01 (0.88, 1.15)	0.94
Q4	540/256,429	1.13 (0.99, 1.28)	0.07
Q5	577/256,237	1.15 (1.01, 1.31)	0.04
**Model 5**			
Q1	449/256,844	1.00 (ref)	
Q2	458/257,043	0.98 (0.86, 1.12)	0.82
Q3	470/256,983	0.96 (0.84, 1.10)	0.56
Q4	540/256,429	1.05 (0.93, 1.20)	0.42
Q5	577/256,237	1.07 (0.93, 1.22)	0.34

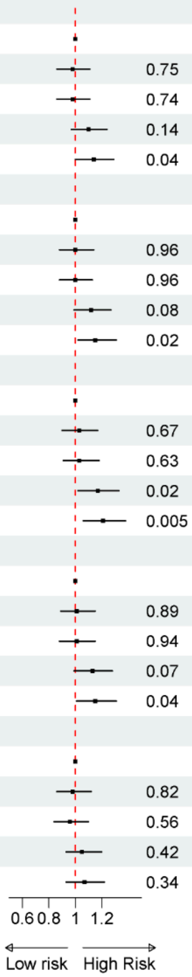

C

Putrescine	Cases/person-years	HR (95%CI)	*p* value
**Model 1**			
Q1	560/255,953	1.00 (ref)	
Q2	458/257,134	0.77 (0.68, 0.87)	<0.001
Q3	462/256,963	0.76 (0.67, 0.86)	<0.001
Q4	451/257,040	0.72 (0.64, 0.82)	<0.001
Q5	563/256,447	0.85 (0.76, 0.96)	0.007
**Model 2**			
Q1	560/255,953	1.00 (ref)	
Q2	458/257,134	0.80 (0.71, 0.90)	<0.001
Q3	462/256,963	0.80 (0.70, 0.90)	<0.001
Q4	451/257,040	0.76 (0.67, 0.86)	<0.001
Q5	563/256,447	0.88 (0.79, 0.99)	0.04
**Model 3**			
Q1	560/255,953	1.00 (ref)	
Q2	458/257,134	0.82 (0.73, 0.93)	0.002
Q3	462/256,963	0.83 (0.73, 0.94)	0.003
Q4	451/257,040	0.80 (0.71, 0.91)	<0.001
Q5	563/256,447	0.94 (0.83, 1.07)	0.35
**Model 4**			
Q1	560/255,953	1.00 (ref)	
Q2	458/257,134	0.81 (0.72, 0.92)	0.001
Q3	462/256,963	0.81 (0.71, 0.92)	0.001
Q4	451/257,040	0.80 (0.70, 0.90)	<0.001
Q5	563/256,447	0.92 (0.82, 1.05)	0.21
**Model 5**			
Q1	560/255,953	1.00 (ref)	
Q2	458/257,134	0.82 (0.73, 0.93)	0.002
Q3	462/256,963	0.82 (0.72, 0.93)	0.002
Q4	451/257,040	0.80 (0.71, 0.91)	<0.001
Q5	563/256,447	0.92 (0.81, 1.04)	0.17

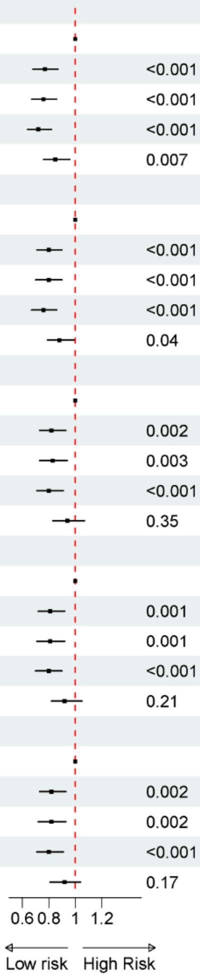

A. The association between dietary spermidine and incident T2DM among participants with two or more diet assessments. B. The association between dietary spermine and incident T2DM among participants with two or more diet assessments. C. The association between dietary putrescine and incident T2DM among participants with two or more diet assessments. Model 1 was adjusted for sociodemographic factors, including age, sex, race/ethnicity, regions. Model 2 was further adjusted for educational level, Townsend index. Model 3 was further adjusted for smoking status, drinking status, physical activity, sleep duration and total energy intake. Model 4 was further adjusted for family history of diabetes, hypertension at baseline, cardiovascular disease at baseline, hyperlipidemia at baseline. Model 5 was further adjusted for BMI group. Abbreviations: HR, hazard ratio. Q1- Q5, quintile 1- quintile 5. BMI, body mass index.

Figure S7. The nonlinear associations of dietary polyamine with incident T2DM among participants with two or more diet assessments

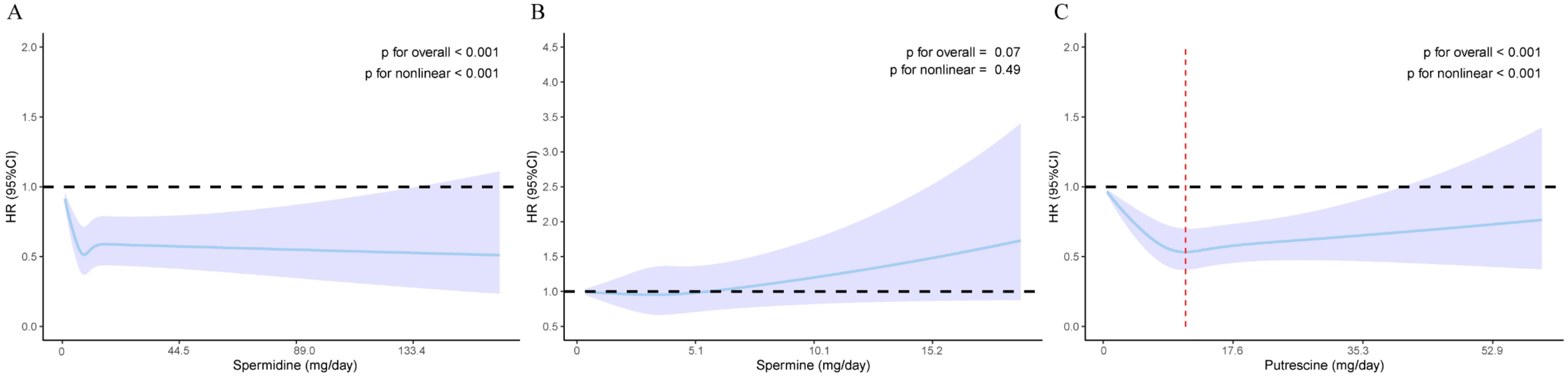

A. The nonlinear association between dietary spermidine and incident T2DM among participants with two or more diet assessments. B. The nonlinear association between dietary spermine and incident T2DM among participants with two or more diet assessments. C. The nonlinear association between dietary putrescine and incident T2DM among participants with two or more diet assessments. Hazard ratio was indicated by solid lines and 95% CIs by shaded areas. The red dashed line indicates the value of dietary spermidine and putrescine at the inflection points. Models were adjusted for age, sex, race/ethnicity, regions, educational level, Townsend index, smoking status, drinking status, physical activity, sleep duration and total energy intake, family history of diabetes, hypertension at baseline, cardiovascular disease at baseline, hyperlipidemia at baseline and BMI group.

Abbreviations: T2DM, type 2 diabetes mellitus. HR, hazard ratio. CI, confidence interval. BMI, body mass index

Figure S8. The associations of dietary polyamine with incident T2DM among participants with two or more years of follow-up

A

Spermidine	Cases/person-years	HR (95%CI)	*p* value
**Model 1**			
Q1	931/411,613	1.00 (ref)	
Q2	760/415,835	0.80 (0.72, 0.88)	<0.001
Q3	774/415,823	0.79 (0.72, 0.87)	<0.001
Q4	795/415,160	0.79 (0.72, 0.87)	<0.001
Q5	962/413,151	0.93 (0.85, 1.02)	0.11
**Model 2**			
Q1	931/411,613	1.00 (ref)	
Q2	760/415,835	0.84 (0.76, 0.92)	<0.001
Q3	774/415,823	0.84 (0.77, 0.93)	<0.001
Q4	795/415,160	0.85 (0.77, 0.93)	<0.001
Q5	962/413,151	0.97 (0.88, 1.06)	0.49
**Model 3**			
Q1	931/411,613	1.00 (ref)	
Q2	760/415,835	0.86 (0.78, 0.95)	0.003
Q3	774/415,823	0.88 (0.79, 0.97)	0.008
Q4	795/415,160	0.88 (0.80, 0.97)	0.01
Q5	962/413,151	1.01 (0.92, 1.12)	0.81
**Model 4**			
Q1	931/411,613	1.00 (ref)	
Q2	760/415,835	0.86 (0.78, 0.95)	0.002
Q3	774/415,823	0.87 (0.79, 0.96)	0.006
Q4	795/415,160	0.88 (0.80, 0.97)	0.01
Q5	962/413,151	0.98 (0.88, 1.08)	0.64
**Model 5**			
Q1	931/411,613	1.00 (ref)	
Q2	760/415,835	0.89 (0.81, 0.98)	0.02
Q3	774/415,823	0.90 (0.82, 0.99)	0.04
Q4	795/415,160	0.91 (0.83, 1.01)	0.07
Q5	962/413,151	0.97 (0.88, 1.08)	0.61

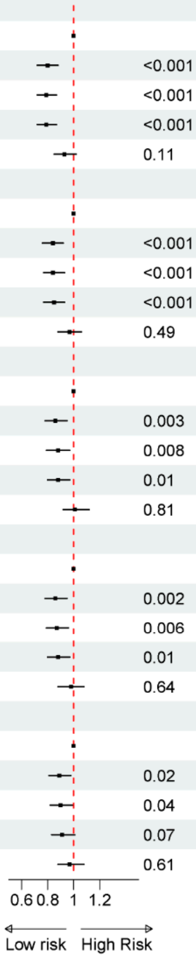

B

Spermine	Cases/person-years	HR (95%CI)	*p* value
**Model 1**			
Q1	449/256,844	1.00 (ref)	
Q2	458/257,043	0.98 (0.86, 1.11)	0.75
Q3	470/256,983	0.98 (0.86, 1.11)	0.74
Q4	540/256,429	1.10 (0.97, 1.24)	0.14
Q5	577/256,237	1.14 (1.00, 1.29)	0.04
**Model 2**			
Q1	449/256,844	1.00 (ref)	
Q2	458/257,043	1.00 (0.88, 1.14)	0.96
Q3	470/256,983	1.00 (0.88, 1.13)	0.96
Q4	540/256,429	1.12 (0.99, 1.27)	0.08
Q5	577/256,237	1.15 (1.02, 1.31)	0.02
**Model 3**			
Q1	449/256,844	1.00 (ref)	
Q2	458/257,043	1.03 (0.90, 1.17)	0.67
Q3	470/256,983	1.03 (0.91, 1.18)	0.63
Q4	540/256,429	1.17 (1.02, 1.33)	0.02
Q5	577/256,237	1.21 (1.06, 1.38)	0.005
**Model 4**			
Q1	449/256,844	1.00 (ref)	
Q2	458/257,043	1.01 (0.89, 1.15)	0.89
Q3	470/256,983	1.01 (0.88, 1.15)	0.94
Q4	540/256,429	1.13 (0.99, 1.28)	0.07
Q5	577/256,237	1.15 (1.01, 1.31)	0.04
**Model 5**			
Q1	449/256,844	1.00 (ref)	
Q2	458/257,043	0.98 (0.86, 1.12)	0.82
Q3	470/256,983	0.96 (0.84, 1.10)	0.56
Q4	540/256,429	1.05 (0.93, 1.20)	0.42
Q5	577/256,237	1.07 (0.93, 1.22)	0.34

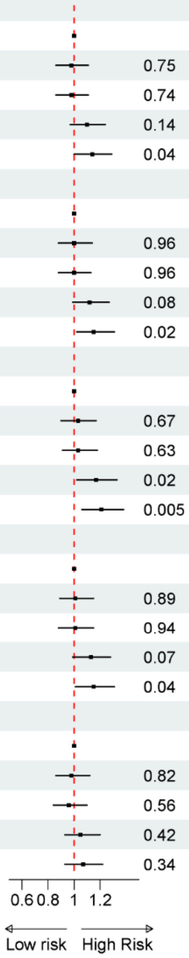

C

Putrescine	Cases/person-years	HR (95%CI)	*p* value
**Model 1**			
Q1	560/255,953	1.00 (ref)	
Q2	458/257,134	0.77 (0.68, 0.87)	<0.001
Q3	462/256,963	0.76 (0.67, 0.86)	<0.001
Q4	451/257,040	0.72 (0.64, 0.82)	<0.001
Q5	563/256,447	0.85 (0.76, 0.96)	0.007
**Model 2**			
Q1	560/255,953	1.00 (ref)	
Q2	458/257,134	0.80 (0.71, 0.90)	<0.001
Q3	462/256,963	0.80 (0.70, 0.90)	<0.001
Q4	451/257,040	0.76 (0.67, 0.86)	<0.001
Q5	563/256,447	0.88 (0.79, 0.99)	0.04
**Model 3**			
Q1	560/255,953	1.00 (ref)	
Q2	458/257,134	0.82 (0.73, 0.93)	0.002
Q3	462/256,963	0.83 (0.73, 0.94)	0.003
Q4	451/257,040	0.80 (0.71, 0.91)	<0.001
Q5	563/256,447	0.94 (0.83, 1.07)	0.35
**Model 4**			
Q1	560/255,953	1.00 (ref)	
Q2	458/257,134	0.81 (0.72, 0.92)	0.001
Q3	462/256,963	0.81 (0.71, 0.92)	0.001
Q4	451/257,040	0.80 (0.70, 0.90)	<0.001
Q5	563/256,447	0.92 (0.82, 1.05)	0.21
**Model 5**			
Q1	560/255,953	1.00 (ref)	
Q2	458/257,134	0.82 (0.73, 0.93)	0.002
Q3	462/256,963	0.82 (0.72, 0.93)	0.002
Q4	451/257,040	0.80 (0.71, 0.91)	<0.001
Q5	563/256,447	0.92 (0.81, 1.04)	0.17

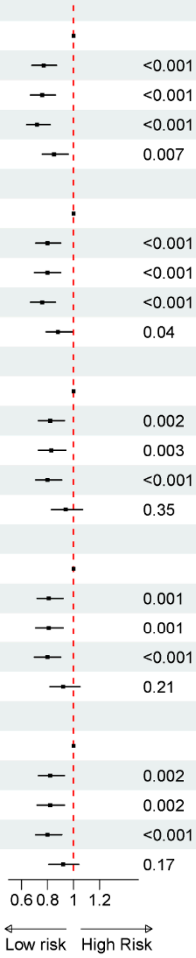

A. The association between dietary spermidine and incident T2DM among participants with two or more years of follow-up. B. The association between dietary spermine and incident T2DM among participants with two or more years of follow-up. C. The association between dietary putrescine and incident T2DM among participants with two or more years of follow-up. Model 1 was adjusted for sociodemographic factors, including age, sex, race/ethnicity, regions. Model 2 was further adjusted for educational level, Townsend index. Model 3 was further adjusted for smoking status, drinking status, physical activity, sleep duration and total energy intake. Model 4 was further adjusted for family history of diabetes, hypertension at baseline, cardiovascular disease at baseline, hyperlipidemia at baseline. Model 5 was further adjusted for BMI group. Abbreviations: HR, hazard ratio. Q1- Q5, quintile 1- quintile 5. BMI, body mass index.

Figure S9. The nonlinear associations of dietary polyamine with incident T2DM among participants with two or more years of follow-up

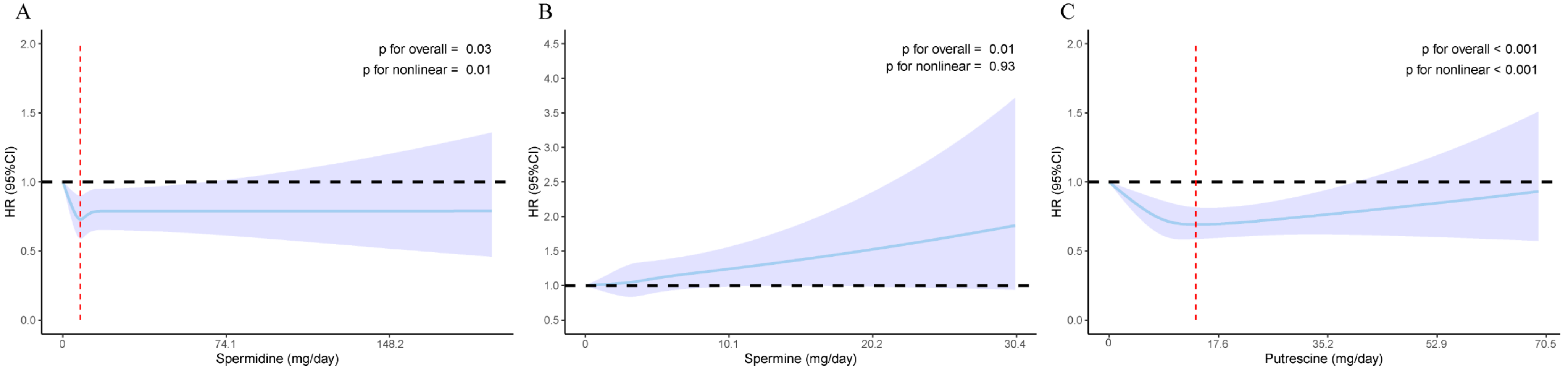

A. The nonlinear association between dietary spermidine and incident T2DM among participants with two or more years of follow-up. B. The nonlinear association between dietary spermine and incident T2DM among participants with two or more years of follow-up. C. The nonlinear association between dietary putrescine and incident T2DM among participants with two or more years of follow-up. Hazard ratio was indicated by solid lines and 95% CIs by shaded areas. The red dashed line indicates the value of dietary spermidine and putrescine at the inflection points. Models were adjusted for age, sex, race/ethnicity, regions, educational level, Townsend index, smoking status, drinking status, physical activity, sleep duration and total energy intake, family history of diabetes, hypertension at baseline, cardiovascular disease at baseline, hyperlipidemia at baseline and BMI group.

Abbreviations: T2DM, type 2 diabetes mellitus. HR, hazard ratio. CI, confidence interval. BMI, body mass index

Figure S10. The associations of dietary polyamine with incident T2DM among participants with five or more years of follow-up

A

Spermidine	Cases/person−years	HR (95%CI)	*p* value
**Model 1**			
Q1	750/410,762	1.00 (ref)	
Q2	625/414,009	0.82 (0.73, 0.91)	<0.001
Q3	621/414,108	0.79 (0.71, 0.88)	<0.001
Q4	641/413,628	0.79 (0.71, 0.88)	<0.001
Q5	779/412,068	0.94 (0.85, 1.04)	0.23
**Model 2**			
Q1	750/410,762	1.00 (ref)	
Q2	625/414,009	0.86 (0.77, 0.96)	0.005
Q3	621/414,108	0.84 (0.76, 0.94)	0.002
Q4	641/413,628	0.85 (0.76, 0.95)	0.003
Q5	779/412,068	0.98 (0.88, 1.08)	0.67
**Model 3**			
Q1	750/410,762	1.00 (ref)	
Q2	625/414,009	0.88 (0.79, 0.98)	0.03
Q3	621/414,108	0.88 (0.79, 0.98)	0.02
Q4	641/413,628	0.89 (0.80, 0.99)	0.04
Q5	779/412,068	1.03 (0.92, 1.15)	0.61
**Model 4**			
Q1	750/410,762	1.00 (ref)	
Q2	625/414,009	0.89 (0.79, 0.99)	0.03
Q3	621/414,108	0.88 (0.79, 0.98)	0.02
Q4	641/413,628	0.89 (0.80, 0.99)	0.04
Q5	779/412,068	0.99 (0.89, 1.11)	0.91
**Model 5**			
Q1	750/410,762	1.00 (ref)	
Q2	625/414,009	0.91 (0.82, 1.02)	0.10
Q3	621/414,108	0.91 (0.81, 1.01)	0.08
Q4	641/413,628	0.92 (0.83, 1.03)	0.15
Q5	779/412,068	0.99 (0.89, 1.11)	0.89

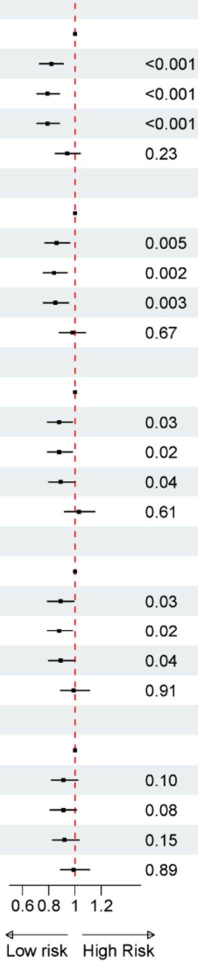

B

Spermine	Cases/person−years	HR (95%CI)	*p* value
**Model 1**			
Q1	616/412,182	1.00 (ref)	
Q2	625/413,636	1.00 (0.89, 1.11)	0.95
Q3	676/413,460	1.06 (0.95, 1.18)	0.33
Q4	709/413,004	1.09 (0.98, 1.21)	0.13
Q5	790/412,292	1.18 (1.06, 1.31)	0.003
**Model 2**			
Q1	616/412,182	1.00 (ref)	
Q2	625/413,636	1.03 (0.92, 1.15)	0.58
Q3	676/413,460	1.10 (0.98, 1.22)	0.10
Q4	709/413,004	1.13 (1.01, 1.26)	0.03
Q5	790/412,292	1.21 (1.09, 1.34)	<0.001
**Model 3**			
Q1	616/412,182	1.00 (ref)	
Q2	625/413,636	1.06 (0.94, 1.18)	0.35
Q3	676/413,460	1.14 (1.02, 1.28)	0.02
Q4	709/413,004	1.18 (1.05, 1.32)	0.004
Q5	790/412,292	1.26 (1.13, 1.41)	<0.001
**Model 4**			
Q1	616/412,182	1.00 (ref)	
Q2	625/413,636	1.04 (0.93, 1.16)	0.51
Q3	676/413,460	1.11 (0.99, 1.24)	0.06
Q4	709/413,004	1.14 (1.02, 1.28)	0.02
Q5	790/412,292	1.21 (1.08, 1.36)	<0.001
**Model 5**			
Q1	616/412,182	1.00 (ref)	
Q2	625/413,636	1.04 (0.93, 1.16)	0.49
Q3	676/413,460	1.09 (0.97, 1.22)	0.14
Q4	709/413,004	1.11 (0.99, 1.24)	0.08
Q5	790/412,292	1.14 (1.02, 1.28)	0.02

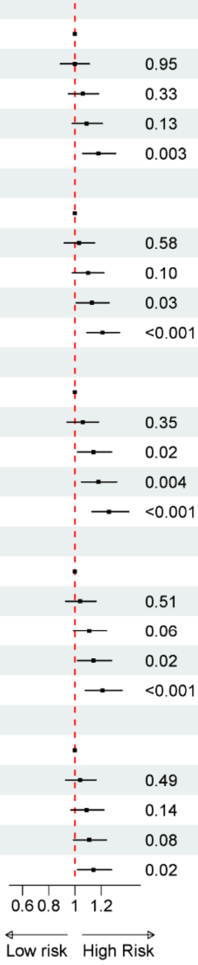

C

Putrescine	Cases/person−years	HR (95%CI)	*p* value
**Model 1**			
Q1	798/411,416	1.00 (ref)	
Q2	632/413,532	0.76 (0.69, 0.85)	<0.001
Q3	625/413,518	0.74 (0.66, 0.82)	<0.001
Q4	631/413,330	0.73 (0.66, 0.81)	<0.001
Q5	730/412,778	0.80 (0.72, 0.88)	<0.001
**Model 2**			
Q1	798/411,416	1.00 (ref)	
Q2	632/413,532	0.80 (0.72, 0.89)	<0.001
Q3	625/413,518	0.78 (0.71, 0.87)	<0.001
Q4	631/413,330	0.78 (0.70, 0.87)	<0.001
Q5	730/412,778	0.84 (0.76, 0.93)	<0.001
**Model 3**			
Q1	798/411,416	1.00 (ref)	
Q2	632/413,532	0.82 (0.73, 0.91)	<0.001
Q3	625/413,518	0.80 (0.72, 0.90)	<0.001
Q4	631/413,330	0.81 (0.73, 0.90)	<0.001
Q5	730/412,778	0.87 (0.79, 0.97)	0.01
**Model 4**			
Q1	798/411,416	1.00 (ref)	
Q2	632/413,532	0.81 (0.73, 0.91)	<0.001
Q3	625/413,518	0.80 (0.72, 0.89)	<0.001
Q4	631/413,330	0.80 (0.72, 0.90)	<0.001
Q5	730/412,778	0.86 (0.77, 0.95)	0.005
**Model 5**			
Q1	798/411,416	1.00 (ref)	
Q2	632/413,532	0.83 (0.75, 0.92)	<0.001
Q3	625/413,518	0.82 (0.74, 0.91)	<0.001
Q4	631/413,330	0.82 (0.74, 0.91)	<0.001
Q5	730/412,778	0.86 (0.77, 0.95)	0.005

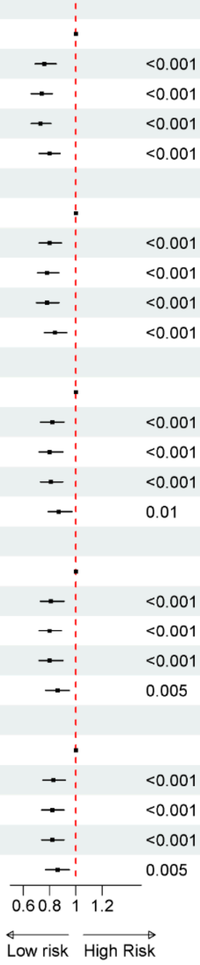

A. The association between dietary spermidine and incident T2DM among participants with five or more years of follow-up. B. The association between dietary spermine and incident T2DM among participants with five or more years of follow-up. C. The association between dietary putrescine and incident T2DM among participants with five or more years of follow-up. Model 1 was adjusted for sociodemographic factors, including age, sex, race/ethnicity, regions. Model 2 was further adjusted for educational level, Townsend index. Model 3 was further adjusted for smoking status, drinking status, physical activity, sleep duration and total energy intake. Model 4 was further adjusted for family history of diabetes, hypertension at baseline, cardiovascular disease at baseline, hyperlipidemia at baseline. Model 5 was further adjusted for BMI group. Abbreviations: HR, hazard ratio. Q1- Q5, quintile 1- quintile 5. BMI, body mass index.

Figure S11. The nonlinear associations of dietary polyamine with incident T2DM among participants with five or more years of follow-up

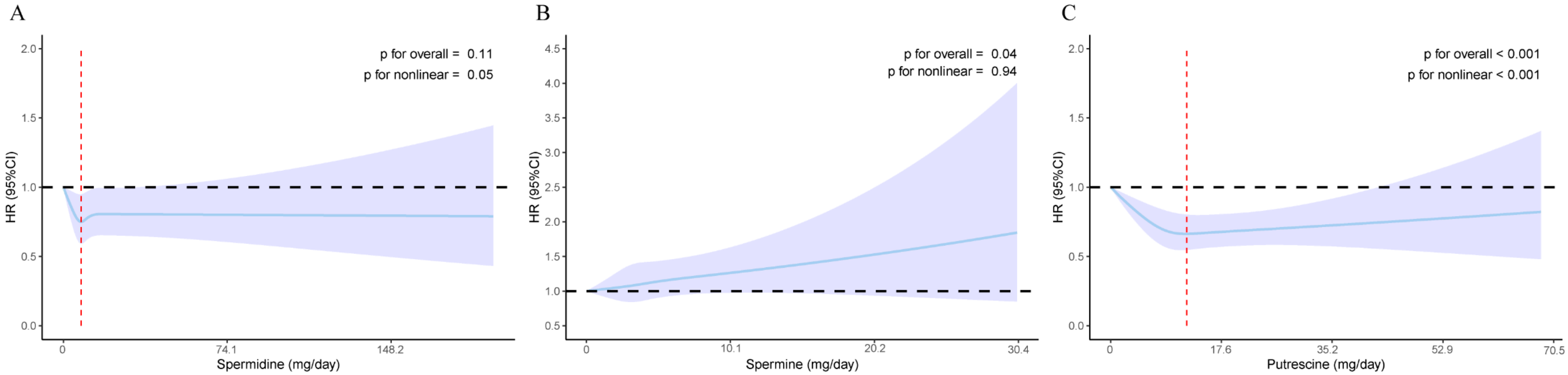

A. The nonlinear association between dietary spermidine and incident T2DM among participants with five or more years of follow-up. B. The nonlinear association between dietary spermine and incident T2DM among participants with five or more years of follow-up. C. The nonlinear association between dietary putrescine and incident T2DM among participants with five or more years of follow-up. Hazard ratio was indicated by solid lines and 95% CIs by shaded areas. The red dashed line indicates the value of dietary spermidine and putrescine at the inflection points. Models were adjusted for age, sex, race/ethnicity, regions, educational level, Townsend index, smoking status, drinking status, physical activity, sleep duration and total energy intake, family history of diabetes, hypertension at baseline, cardiovascular disease at baseline, hyperlipidemia at baseline and BMI group.

Abbreviations: T2DM, type 2 diabetes mellitus. HR, hazard ratio. CI, confidence interval. BMI, body mass index

Table S6. Nutrient Database for Polyamine Intake*

Food Category	Spermidine	Spermine	Putrescine	Reference
		Drinks		
Water	0	0	0	
Cola	BDL	BDL	BDL	[44]
Squash	0.7	0.3	28.4	[10]
Orange soft drink	0.7	0.3	28.4	[10]
Orange juice	6	BDL	159.8	[45]
Grapefruit juice	6	BDL	99.2	[45]
Pure fruit/vegetable juice	6.8	1.0	15.9	[46]
Apple juice	2	BDL	11.1	[46]
Grape juice	5.1	BDL	9	[46]
Apricot juice	7.0	BDL	8	[46]
Tomato juice	13.2	1	35.5	[46]
Fruit smoothie	NA	NA	NA	
Dairy smoothie	NA	NA	NA	
Coffee	BDL	BDL	BDL	[45]
Tea	BDL	BDL	BDL	[45]
		Other non-alcoholic drinks		
Full cream milk	2	2	1	[47]
Semi-skimmed milk	0.4	0.4	0.3	[45]
Skimmed milk	BDL	BDL	BDL	[45]
Powdered milk	7	7	1.5	[45]
Soybean milk	112	14	24	[44]
Goat/sheep milk	21.0	2.8	3.1	[10]
Ovine milk	2.1	2.5	0.5	[10]
Caprine milk	39.9	3.0	5.7	[10]
Other type of milk	3	6	0.5	[44]
Human milk	3	6	0.5	[44]
Hot chocolate	21.5	3	9	[45]
Other drink	NA			
Barley cup	NA	NA	NA	
Other mixed fruit drink	NA	NA	NA	
		Alcohol		
Red wine	2	BDL	39.1	[45]
Rose wine	0.7	BDL	68.1	[48]
White wine	1	BDL	9.0	[45]
Beer/cider	BDL	BDL	21.2	[45]
Fortified wine	1	BDL	BDL	[46]
Cognac	1	BDL	BDL	[46]
Spirits	1	BDL	BDL	[46]
Whisky	1	BDL	BDL	[46]
Other alcohol	BDL	BDL	35.4	[46]
Liqueur wine	BDL	BDL	35.4	[46]

Breakfast cereal				
Breakfast cereals, mixed	166.6	31.6	113.4	[11]
Porridge	BDL	BDL	BDL	[45]
Bran cereal	351.1	438.9	553.6	[11]
Whole-wheat cereal	168	40	8	[49]
Breads	78.8	17.2	29.6	[46]
White bread	54.2	13.2	13.0	[46]
Oat bread	65.4	12.6	22.1	[46]
Rye bread	86.1	20.7	42.5	[46]
Whole bread	109.3	22.4	40.6	[46]
Oatcakes	65.4	12.6	22.1	[45]
Pastry	BDL	BDL	BDL	[52]
Crumble	NA	NA	NA	
Pizza	35.1	6.0	13	[45]
Pancake	5	1	BDL	[45]
Yorkshire pudding	NA	NA	NA	
Indian snacks	NA	NA	NA	
Croissant	NA	NA	NA	
Scone	NA	NA	NA	
Yogurt/ice-cream				
Yogurt	1	1	BDL	[45]
Ice-cream	BDL	BDL	BDL	[50]
Dessert				
Milk-based pudding	NA	NA	NA	
Other milk-based pudding	NA	NA	NA	
Soya dessert	NA	NA	NA	
Cake	6	1	3	[45]
Doughnut	NA	NA	NA	
Sponge pudding	20	2	10	[51]
Cheesecake	NA	NA	NA	
Other dessert	11.1	2	4	[45]
Strawberry pie	13.2	3	5	[45]
Lemon pie	9	1	3	[45]
Sweet snack				
Milk chocolate	6	1	3	[45]
Dark chocolate	21.5	3	9	[45]
Raisin	3	1	2	[45]
Chocolate	17	7	4	[52]
Sweets	0	0	0	[52]
Sweet biscuits	6	1	2	[45]
Cereal bar	NA	NA	NA	
Savoury snack				
Peanuts	388.7	34.6	61.4	[45]
Nuts	163.2	133.4	57.9	[44, 45, 53]

Almonds	207.4	54.2	35.6	[45]
Cashews	124	363	45	[44]
Pistachio	208.6	54.3	87.4	[45]
Walnuts	112.9	61.9	63.5	[53]
Seeds	225.4	64.3	57.9	[44, 45]
Sesame	126	22	29	[44]
Hazelnut	144.5	32.3	47.7	[45]
Sunflower seed	383	89	34	[44]
Pumpkin seed	248	114	121	[44]
Crisp	13	245	171	[44]
Biscuits	6	1	2	[45]
Olives	NA	NA	NA	
Other savoury snack	NA	NA	NA	
Soup	95.4	22.0	31.8	[45]
Vegetable soup	35.7	5	24.5	[45]
Lentil soup	151.5	36.5	38.5	[54]
Starchy food				
Pasta	35.1	6	13	[45]
White rice	BDL	BDL	1	[45]
Brown rice	44.1	49.4	56.7	[11]
Sushi	NA	NA	NA	
Snack pot	NA	NA	NA	
Couscous	NA	NA	NA	
Other grain	42.1	29.5	34.0	[44, 45]
Barley	16	20	17	[44]
Semolina	5.0	23.1	25.1	[45]
Soft wheat	105.3	45.4	60.0	[45]
Cheese				
Cheese	1.4	0.1	0.4	[45]
Hard cheese	53.7	17.3	987.0	[11]
Soft cheese	1.4	0.1	1.7	[11]
Blue cheese	111.5	7.4	475.3	[11]
Cottage cheese	NA	NA	NA	
Mozzarella	BDL	BDL	BDL	[44]
Goat's cheese	4.2	1.6	7.2	[45]
Egg				
Whole egg	BDL	BDL	20.5 ± 0.1	[45]
Scotch egg	BDL	BDL	20.5 ± 0.1	[45]
Omelette	NA	NA	NA	
Other egg	NA	NA	NA	
Meat				
Sausage	20.0	46.8	211.0	[45]
Toulouse sausage	16.3	46.2	3.0	[45]
Spicy sausage (merguez)	12.2	48.3	8.1	[45]

Frankfurter sausage	27.0	31.1	11.1	[45]
Garlic sausage	31.5	45.5	7	[45]
Rosette (gamy pork sausage)	13.0	62.9	1025.6	[45]
Beef	17.5	140.8	30.5	[45]
Pork	9.3	72.3	4	[45]
Lamb	39.7	131.3	8.2	[45]
Poultry	91.1	65.6	168.0	[10, 45]
Chicken	78.2	61.4	10.1	[45]
Turkey	103.9	69.8	325.8	[10, 45]
Chicken skin	78.5	120.1	0.0	[10]
Bacon	6	26.2	2	[45]
Ham	8.1	63.4	5	[45]
Liver	199.7	509.0	26.1	[48]
Other meat	82	305	135.5	[44, 55]
Duck	58	323	20	[44]
Game	106	287	251	[55]
Fish				
Oily fish	45.1	47.6	35.7	[44,45]
Salmon	60.0	45.0	44.0	[45]
Mackerel	49	92	20.0	
Herring	26.2	5.9	43.1	
Shrimp	2.4	BDL	2.7	[45]
Lobster/crab	4.0	BDL	1	[45]
Shellfish	93.6	64.6	261.6	[45]
Scallops (coral)	14.5	49.9	487.3	[45]
Scallops (white)	6.8	14.0	285.7	[45]
Muscles	259.5	129.8	11.7	[45]
Other fish	19.8	51.0	10.2	[44, 11]
Trout	27.5	44.0	20.4	[11]
Squid	12	58	BDL	[44]
Tinned tuna	6	10	2	[45]
White fish	160	75	119	[44]
Vegetarian alternatives				
Vegetarian sausages/burgers	NA	NA	NA	
Tofu	107	32	20	[44]
Quorn	NA	NA	NA	
Other vegetarian alternative	134.1	23.9	30.8	[45]
Nut roast	268.2	47.7	61.5	[45]
Almond (grilled nut)	207.4	54.2	35.6	[45]
Pistachio (grilled nut)	208.6	54.3	87.4	[45]
Peanut (grilled nut)	388.7	34.6	61.4	[45]
Falafel	NA	NA	NA	
Vegetable				

Cooked bean	57.1	24.7	55.6	[11]
Pulses	165.4	45.7	30.7	[45, 55]
Kidney beans	134	120	4	[55]
Chick peas	198.3	6	29.4	[45]
Lentils	163.9	11.1	58.7	[45]
Cooked potatoes	105.0	26.0	245.0	[47]
Butter	BDL	BDL	BDL	[45]
Potato	77.0	15.0	110.0	[47]
Mashed potato	120.7	2	87.4	[45]
Mixed vegetable	106.3	14.0	39.1	[45]
Cabbage	195.3	18.1	74.9	[45]
Avocado	12.7	6.6	3.1	[45]
Broad bean	495.7	118.6	203.1	[53]
Green bean	74.4	13.3	116.8	[53]
Beetroot	29.1	BDL	51.7	[45]
Broccoli	214.0	22.8	64.2	[45]
Cabbage/kale	195.3	8.1	74.9	[45]
Carrot	55.0	12.0	17.0	[47]
Cauliflower	56.0	43.0	171.0	[47]
Celery	22.0	0.7	25.0	[45]
Courgette	107.0	7.5	393.5	[45]
Cucumber	65.0	1.3	98.9	[45]
Garlic	155.3	33.8	13.1	[45]
Leek	104.7	10.7	27.4	[45]
Leek (green)	127.2	12.3	30.2	[45]
Leek (white)	82.2	9.1	24.6	[45]
Lettuce	95.6	5.9	89.4	[10,45]
Mushroom	450.0	3.0	4.0	[45]
Onion	35.45	BDL	5.7	[45]
Radish	87.6	1.7	10.5	[45]
Pea	348.4	19.1	196.5	[45]
Sweet pepper	32.4	8.4	26.9	[45]
Spinach	185	18	50	[44]
Sprouts	127.6	9.1	59.1	[45]
Corn	298	6	520	[44]
Sweet potato	20	29	15	[44]
Fresh tomato	19.4	BDL	381.1	[45]
Tinned tomato	34.2	1	163.1	[45]
Turnip	405	19	219	[44]
Watercress	201	7	20	[44]
Other vegetables	82.7	48.7	116.7	[44, 52]
Aubergine	86	27	214	[44]
Asparagus	120	28	61	[52]
Pumpkin	42	91	75	[52]

Stewed fruit				
Apple stew	7	BDL	5	[45]
Prune	10.1	2	6.1	[45]
Dried fruit	8.0	0.7	15.4	[44, 45]
Raisins	3	1	2	[45]
Dates	10.1	1	32.2	[45]
Prune (dried)	11	0	12	[44]
Mixed fruit	15.3	1	27.6	[45]
Apple	5-17	0	12	[47]
Banana	44.9	1	317.3	[45]
Berry	37.7	6.9	14.8	[44, 45]
Strawberry	40.3	13.7	18.5	[45]
Blueberry	35	BDL	11	[44]
Cherry	19	BDL	53	[44]
Grapefruit	19.5	1.5	436	[44]
White grapefruit	19	3	292	[44]
Ruby grapefruit	20	BDL	580	[44]
Grape	22.5	1.6	26.3	[45]
Grape(red)	15.2	0.1	34.2	[45]
Grape(green)	29.8	3.1	18.3	[45]
Mango	206.5	15.8	907.5	[11]
Melon	77.7	4.9	22.7	[45]
Orange	17.8	BDL	1047.7	[45]
Satsuma	80	BDL	860	[44]
Peach	18.4	BDL	7.4	[45]
Pear	18.4	0.2	0.4	[45]
Pineapple	27.0	10.9	7.6	[45]
Plum	11.7	0.5	30.6	[53]
Other fruit	31.2	1.7	20.0	[44, 45, 53]
Kiwi	37.3	7.4	13.3	[45]
Papaya	37	BDL	53	[44]
Pomegranate	5	BDL	2	[44]
Fig	36	BDL	25	[44]
Apricot	40.6	1.0	6.8	[53]
Seasonings				
Salt	BDL	BDL	BDL	[45]
Sugar	0	0	0	[52]
Artificial sweetener	NA	NA	NA	

*Values expressed in mean ± SD (nmol/g or ml); BDL, below detection limits; NA, not available.
